# Integrated analysis of miRNA profiles and gut bacterial changes in *Altica viridicyanea* following antibiotic treatment

**DOI:** 10.1002/ece3.10660

**Published:** 2023-10-31

**Authors:** Yipeng Ren, Yuan Wang, Juhong Chen, Siying Fu, Wenjun Bu, Huaijun Xue

**Affiliations:** ^1^ Institute of Entomology, College of Life Sciences Nankai University Tianjin China

**Keywords:** adaptive evolution, *Altica viridicyanea*, antibiotic treatment, gut bacteria, microRNAs

## Abstract

The gut bacteria involves in insect homeostasis by playing essential roles in host physiology, metabolism, innate immunity, and so forth. microRNAs (miRNAs) are endogenous small noncoding RNAs that posttranscriptionally regulate gene expression to affect immune or metabolic processes in insects. For several non‐model insects, the available knowledge on the relationship between changes in the gut bacteria and miRNA profiles is limited. In this study, we investigated the gut bacterial diversity, composition, and function from *Altica viridicyanea* feeding on normal‐ and antibiotic‐treated host plants using 16S rRNA amplicon sequencing; antibiotics have been shown to affect the body weight and development time in *A. viridicyanea*, suggesting that the gut bacteria of the normal sample were more diverse and abundant than those of the antibiotic‐fed group, and most of them were involved in various physical functions by enrichment analysis. Furthermore, we executed small RNA transcriptome sequencing using the two experimental groups to obtain numerous sRNAs, such as piRNAs, siRNAs, and known and novel miRNAs, by data mapping and quality control, and furthermore, a total of 224 miRNAs were identified as significantly differentially expressed miRNAs, of which some DEMs and their target genes participated in immune‐ and metabolism‐related pathways based on GO and KEGG annotation. Besides, regarding the regulatory roles of miRNA and target genes, a interaction network of DEM‐target gene pairs from eight immune‐ or metabolism‐related signaling pathways were constructed. Finally, we discovered that DEMs from above pathways were significantly positively or negatively correlated with gut bacterial alterations following antibiotic treatment. Collectively, the observations of this study expand our understanding of how the disturbance of gut bacteria affects miRNA profiles in *A. viridicyanea* and provide new valuable resources from extreme ranges for future studies on the adaptive evolution in insects.

## Graphical abstract

This finding provides the first genome‐wide identification and characterization of miRNAs and investigates the changes in gut bacterial microbiota in *Altica viridicyanea* under antibiotic‐reared condition. The disturbance of gut bacterial microbiota affects miRNA profiles of *A. viridicyane*a feeding on antibiotic‐treated plants, which can lay the foundation for uncovering the adaptive evolution between gut bacteria–host interactions in Coleoptera.

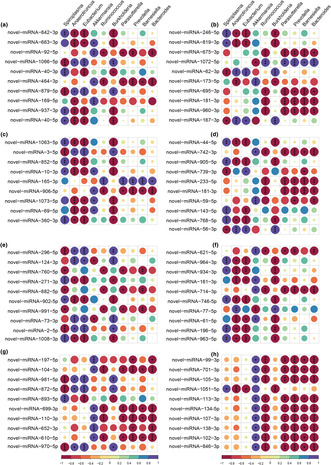

## 1 INTRODUCTION

microRNAs (miRNAs), serving as important regulators of gene expression, are a type of small noncoding RNA (sncRNA) with a length of approximately 18–24 nucleotides (nt) that are involved in a variety of biological processes, such as growth, development, apoptosis, and innate immunity, by promoting mRNA degradation or inhibiting translation to regulate the expression of target genes at the posttranscriptional level (Bartel, 2004; Zhao et al., 2022). With the development of next‐generation sequencing technology, it has become an essential tool to explore the miRNA profiles and their functional prediction through RNA sequencing technology, which provides an accessible opportunity to overall screen the differential expression of the genome at the transcriptional level (Wolf, 2013). To date, a growing number of studies have shown the functional roles of miRNAs in insects. For instance, miR‐8, miR‐308, miR‐958, miR‐964, and miR‐317 appear to negatively regulate the Toll and Imd signaling pathway in *Drosophila* (Choi & Hyun, 2012; Lee & Hyun, 2014; Li et al., 2019; Li, Li, et al., 2017; Li, Xu, et al., 2017; Yao et al., 2023). In *Bombyx mori* (mulberry silkworm) larvae, miR‐278‐3p, interacting with *IBP2* (insulin‐related peptide binding protein 2), positively initiates the mRNA expression of cytoplasmic polyhedrosis virus (Wu, Qin, et al., 2016). It was shown that the JNK (Jun N‐terminal kinase) pathway plays a key role in the immune system by regulating miR‐184 in bacteria‐infected pea aphids (*Acyrthosiphon pisum*; Ma et al., 2020). Furthermore, the miR‐n58/n174 cluster expression level was highly expressed in female white‐backed planthopper, *Sogatella furcifera*, and meanwhile, the target gene, E3 ubiquitin‐protein ligase RNF123, decreased in the females, as a sex‐biased functional miRNA (Chang et al., 2018). In addition, it has been confirmed that *Drosophila* miR‐92a/92b plays vital roles in the modulation of PDF neuronal excitability and maintenance of a neuroblast pool (Chen & Rosbash, 2017; Yuva‐Aydemir et al., 2015).

The gut microbiota, such as bacteria, archaea, and eukaryotes, can produce beneficial components related to strengthening the immune system, defending against pathogens, and regulating host metabolism (Sekirov et al., 2010; Thursby & Juge, 2017). In herbivorous insects, symbiotic bacterial communities have likely evolved to favor species by detoxifying or resisting the toxins liberated in host guts, which is known as the gut microbial facilitation hypothesis (Hammer & Bowers, 2015). There is a report on the investigations of gut microbiome between the two *Apriona* species with host niche competition, suggesting that they were annotated into degrading plant toxic secondary compounds based on KEGG enrichment (Zhang, Wang, et al., 2022). Additionally, it is shown that the gut bacteria facilitate adaptation to crop rotation in the western corn rootworm (Chu et al., 2013), and they are involved in degrading defense chemicals consumed in various pests (Wei et al., 2020). Regarding the improving resistance against pathogens, in a hemipteran pest, *Riptortus pedestris*, the knockdown of the *thanatin* gene significantly increased the population of *Burkholderia*, a gut bacteria in the M4 crypt, in response to *Escherichia coli* K12 injection (Lee et al., 2022). The mortality of samples from a non‐axenic *Lymantria dispar asiatica* larval gut was significantly higher than that of axenic larvae, and gut bacteria could be translocated from the gut to the hemocoel in the non‐axenic group under *Beauveria bassiana* infection (Bai et al., 2023). To the best of knowledge, it is well known that the disturbance of microbiome homeostasis, known as “dysbiosis,” resulted in an influence on physiological parameters within the immune systems or metabolic levels of host, owing to changes in the microbial community functionality (Egan & Gardiner, 2016; Legrand et al., 2020). In recent year, the integrated analysis of multiomics data is available on the host–gut microbiota interactions in non‐model dysbiosis insects. For example, antibiotic exposure disturbs the gut bacteria in *Apis mellifera*, and then influences host phenotypes, olfactory learning, and memory abilities using field or laboratory‐generated bees combined with metagenomic sequencing, RNA sequencing, and proteome and metabolomics analysis (Zhang, Mu, et al., 2022). A recent study indicated that the gut bacteria of *Plagiodera versicolora* is an essential determinant of nutritional metabolism and immunity using comparative transcriptomic analysis and 16S rRNA amplicon sequencing of gut and body tissues in axenic and non‐axenic larvae (Ma et al., 2021). Besides, Xie and colleagues observed miRNA and mRNA changes and constructed the miRNA–gene regulatory network under gut microbiota depletion using the abdomens of female *Bactrocera dorsalis* through RNA transcriptome and 16S rRNA amplicon sequencing (Xie et al., 2023). On the basis of the above observations, elucidating the disturbance of gut bacteria how to affect the target genes through regulation of miRNA expression will help us to deeply understand the adaptive evolution and maintaining homeostasis of insect populations.

*Altica viridicyanea*, feeding on *Geranium nepalense* Sweet (Geraniaceae), is a species of *Altica* Geoffroy (Coleoptera: Chrysomelidae) flea beetles and serves as a well‐known model of ecological speciation (Reid & Beatson, 2015; Xue et al., 2011, 2014). It is easy to keep under laboratory conditions for investigating its physiological and behavioral changes, and its genomic data have been published (Xue et al., 2009, 2016). The gut microbiota is an essential determinant of adaptive evolution in *A. viridicyanea*, but there is little evidence on the interplay between gut bacteria and immune or metabolism‐related miRNAs in *A. viridicyanea* combined with 16S rRNA amplicon and small RNA sequencing (sRNA‐seq). In this study, we first used 16S rRNA amplicon sequencing by the Illumina HiSeq 2000 platform to detect the differences in the diversity and composition of gut bacterial communities in *A. viridicyanea* feeding on normal‐ or antibiotic‐treated host plants. Furthermore, sRNA‐seq analysis among above two experimental groups was performed on the *A. viridicyanea* guts to explore the characterization, expression patterns, and functional and regulatory roles of miRNAs. Lastly, a set of critical immune and metabolic differentially expressed miRNAs (DEMs) were selected, and the interaction relationships between DEMs and gut bacterial changes were executed based on bioinformatic prediction and Pearson correlation analysis. Taken together, this study can increase our understanding of the regulatory mechanisms of miRNAs and target genes in the insect gut and offer scientific data for future researches on the interaction between immunity or metabolism and gut bacteria in Coleoptera.

## 2 MATERIALS AND METHODS

### 2.1 Insect rearing and treatment

Approximately 40 adults of *A. viridicyanea* were collected from wild populations in Jizhou district (40.22′ N, 117.50′ E), Tianjin, P.R. China on May 19, 2022. All beetles were reared in a growth chamber held at 25°C with 60% relative humidity (RH) and long‐day lighting conditions (16 h Light, 8 h Dark) and allowed to feed on its host plant *G. nepalense*, which was collected at the same location as insect samples. Next, newly born eggs and hatched larvae of *A. viridicyanea* were collected and kept in dishes with moist filter papers under above rearing conditions. The molted 2nd‐instar larvae were fed normal host leaves or host leaves completely submerged in antibiotic solution (50 μg/mL tetracycline, 200 μg/mL rifampicin, and 100 μg/mL streptomycin = 1:2:4) for 5 min, until 10 days after adult emerging, which were sexually mature adults (Wei et al., 2020). Furthermore, a total number of 80 normal‐ (control group, C) and 80 antibiotic‐fed (antibiotic group, A) samples were starved for 2 days, and later, all whole gut tissues were gently dissected by clamping the head of live beetles with sterile forceps and removing the head. Finally, the gut tissues from 10 individuals of each experimental treatment were surface sterilized in 70% ethanol for 1 min and randomly pooled to construct the 16S rRNA or sRNA‐seq libraries as one biological replicate. All samples were flash frozen in liquid nitrogen and stored at −80°C until subsequent experiments. In this research, all animal experimental procedures were performed and approved by the Animal Care and Use Committee of Nankai University.

### 2.2 DNA extraction and 16S rRNA amplicon sequencing

A total of three biological replications from each treatment (*n* = 10 per replication) were used to extract DNA with a Genomic DNA extraction Kit (QIAamp) following the manufacturer's instructions. Furthermore, the amount, purity, and integrity of each DNA sample were estimated by a NanoDrop 2100 (Thermo) and electrophoresis of a 1.0% agarose gel, respectively. The results showed a single bright band on the gel of each DNA sample of approximately 20,000 bp (base pairs), and the absorbance ratio of A_260_/A_280_ nm of all DNA samples ranging from 1.8 to 2.0 implied accessible DNA sample quality. Later on, the prepared DNA samples were diluted to 30 ng/μL with sterile water for 16S rRNA amplicon sequencing. In brief, all samples were amplified with the same cycle numbers to ensure the accuracy and dependability of library construction using the barcoded primers V338F (5′‐ACTCCTACGGGAGGCAGCAG‐3′) and V806R (5′‐CGACTACHVGGGTWTCTAAT‐3′). Moreover, a QuantiFluorTM‐ST blue fluorescence quantitative instrument was used to quantitatively detect the PCR (polymerase chain reaction) products. Next, the efficiency of the above PCR products was tested with 1.2% agarose gel electrophoresis, and then, they were purified with Agencourt AMPure XP beads (Beckman). The purified 16S rRNA amplicons were ligated with Illumina adapters (TruSeq DNA LT Sample Prep Kit), and the produced fragments with adapters were pooled in equimolar amounts. Finally, all the sequencing libraries from the above DNA products were built and sequenced with the Illumina HiSeq 2000 platform according to standard protocols at Beijing Genomics Institute (BGI) Technology Co., Ltd.

### 2.3 Data analysis of 16S rRNA amplicon sequencing

In this study, we used FLASH (version 1.2.11) to filter the raw data for quality check and splice the paired‐end sequences. Next, operational taxonomic units (OTUs) with sequence similarity were clustered through USEARCH (version 7.0.1090) software to discard chimeric sequences. RDP classifier (version 11.5) using the Bayesian algorithm conducted the taxonomic analysis based on the representative sequences of OTUs with a 97% similarity level using the I‐sanger cloud data analysis platform. In addition, alpha diversity was estimated by QIIME (version 1.9.1) workflow (Caporaso et al., 2010), whose results presented the richness of gut bacterial communities by the Chao estimator. Besides, the ACE (abundance‐based coverage estimator), Shannon and Simpson indices were calculated using Mothur (version 1.30.2) to reveal the diversity of gut bacterial community (Li, Wu, et al., 2021). The PCoA (principal coordinate analysis) based on Bray–Curtis dissimilarities was used to identify differences among gut bacterial communities. Collectively, the community heatmap was analyzed at the genus level, and Wilcox Test was applied to estimate the differences between the C and A groups. The functional annotation of bacterial OTUs was performed using Phylogenetic Investigation of Communities by Reconstruction of Unobserved States 2 (PICRUSt2), including COG (Clusters of orthologous groups of proteins), KEGG (Kyoto encyclopedia of genes and genomes), and Metacyc (Metabolic pathways from all domains of life) enrichment analyses (Douglas, 2015; Edgar, 2013). Finally, linear discriminant analysis effect size (LEfSe) was employed to identify biomarkers in different experimental groups (Segata et al., 2011).

### 2.4 Total RNA extraction and sRNA‐seq library construction

A total of three biological replications from each treatment (*n* = 10 per replication) were used to extract total RNA with TRIzol reagent (Invitrogen) according to the manufacturer's procedures. Moreover, the integrity, purity, and concentration of each RNA sample were estimated and assessed by 1.2% agarose gel electrophoresis and the OD_260_/OD_280_ ratio by an Agilent 2100 Bioanalyzer system (Agilent Technologies), respectively. In addition, remaining RNA after library construction was used to qRT‐PCR validation.

In the current study, for small RNA library construction, we built the sRNA‐seq libraries from the qualified RNA using TruSeq Small RNA Sample Prep Kits according to the manufacturer's instructions. In brief, 1 μg of prepared total RNA was ligated to the 5′ and 3′ TruSeq adaptors and reversely transcribed by PCR. Next, the cDNA products were purified and selected from the 18 to 30 nt small RNA fragments by gel electrophoresis. The final constructed libraries were sequenced using 140–160 bp single‐end reads on the DNBSEQ‐G500 platform at Beijing Genomics Institution Technology Co., Ltd.

### 2.5 Data assembly and filtration

Adaptor contamination, low‐quality reads, and undetermined data were removed from the raw reads using SOAPnuke (version 1.5.0) software. Furthermore, the quality of clean reads was estimated by FastQC (version 0.10.1), and the information of Q20 and Q30, representing effective sequencing values, were statistically calculated. Then, we used Bowtie (version 1.1.2) to map clean data to the reference genome of *A. viridicyanea* (Langmead et al., 2009). The mapped reads of each group were assembled by StringTie (version 1.3.0), and finally, the sRNA‐seq data were produced (Pertea et al., 2015).

### 2.6 Identification of sncRNAs

ACGT101‐miR (version 4.2) (LC Sciences) was employed to filter out adapter dimers, junk, low complexity data, and common sRNA contaminants, including ribosomal RNA (rRNA), transfer RNA (tRNA), small nuclear RNA (snRNA), small nucleolar RNA (snoRNA), and repeat sequences, from raw data. The remaining sRNAs were subsequently mapped to specific species precursors with miRbase (version 22.0), RNAcentral (version 16.0), and Rfam (version 13.0). In addition, we used miRbase (version 22.0) and miRDeep2 (version 0.1.3) in a BLAST (basic local alignment search tool) search to identify known or novel miRNAs (Kozomara et al., 2019), and meanwhile, Piano and PHASIS (version 3.3) were used to predict piRNAs and siRNAs, respectively.

### 2.7 Expression analysis of miRNAs

To characterize the expression patterns of identified miRNAs, the expression profiles were calculated and normalized to transcripts per million (TPM), and next, the correlation and principal component analysis (PCA) analyses were performed among all sRNA‐seq libraries based on RPKM (Reads Per Kilobase of exon model per Million mapped reads) using StringTie (version 1.3.0) software and DESeq R (version 1.10.1) package, respectively (Pertea et al., 2015). The Bonferroni correction was used to adjust the *p* values in order to decrease FDR (false discovery rates, *q* values). The significantly differentially expressed miRNAs were those with absolute log_2_ | fold change | >1 and *p* value <.001, calculated by edgeR package (version 3.14.0; Robinson et al., 2010).

### 2.8 Target prediction and functional annotation

To further predict the target genes, RNAhybrid (version 0.1) and miRanda (version 3.3a) were applied to miRNA target prediction by identifying miRNA‐binding sites with computational target prediction algorithms for both combined and overlapping genes (Enright et al., 2003; Lewis et al., 2005). Overlapping genes from the two algorithms were considered target genes. Moreover, to obtain annotation information on the functions of DEMs and target genes, we applied GO (Gene Ontology) and KEGG databases to functional enrichment analysis (Qu et al., 2023). Finally, we constructed a regulatory network of DEM‐target gene axis from eight immune‐ and metabolism‐related signaling pathways with Cytoscape software (version 3.9.1) based on the target and functional prediction (Otasek et al., 2019).

### 2.9 Correlation analysis of DEMs and gut bacterial changes

In order to explore the relationships between sRNA‐seq and 16S rRNA amplicon sequencing data, the Pearson correlation coefficients were calculated for the integrated analysis of the above sequencing results. Briefly, DEMs from eight signaling pathways and the top 10 changes in relative abundance of gut bacteria were selected to construct the heatmaps using R packages (version 2.15.3). Statistical significance was set at *p* values (*p < .05, **p < .01 and ***p < .001). In addition, raw reads have been submitted to the NCBI database under accession number PRJNA946034 and PRJNA946198.

### 2.10 Quantitative real‐time PCR validation and statistical analysis

We randomly selected seven DEMs to validate the sRNA‐seq results using quantitative real‐time PCR (qRT‐PCR). cDNA samples were synthesized from remanent total RNA from the Section 2.4 with TransScript® miRNA First‐Strand cDNA Synthesis SuperMix (TransGen Biotech). Furthermore, qRT‐PCR analysis was performed using TransScript® Green miRNA Two‐Step RT‐qPCR SuperMix (TransGen Biotech) on a CFX96 real‐time PCR Detection System (Bio‐Rad), and the following thermal cycling conditions were described as our previous study (Ren et al., 2023). The snRNA U6 was served as an internal reference gene, and other primers were designed by Primer Premier 5 (Table S9). All reactions were performed in triplicate, and the relative expression was calculated using the $2^{-\Delta \Delta c_{t}}$ method (Livak & Schmittgen, 2001). Finally, the column chart of relative expression of qRT‐PCR and sRNA‐Seq data was produced using GraphPad Prism (version 9.0; GraphPad Software Inc.).

## 3 RESULTS

### 3.1 Gut bacteria in *A. viridicyanea* under antibiotic feeding

**FIGURE 1:**
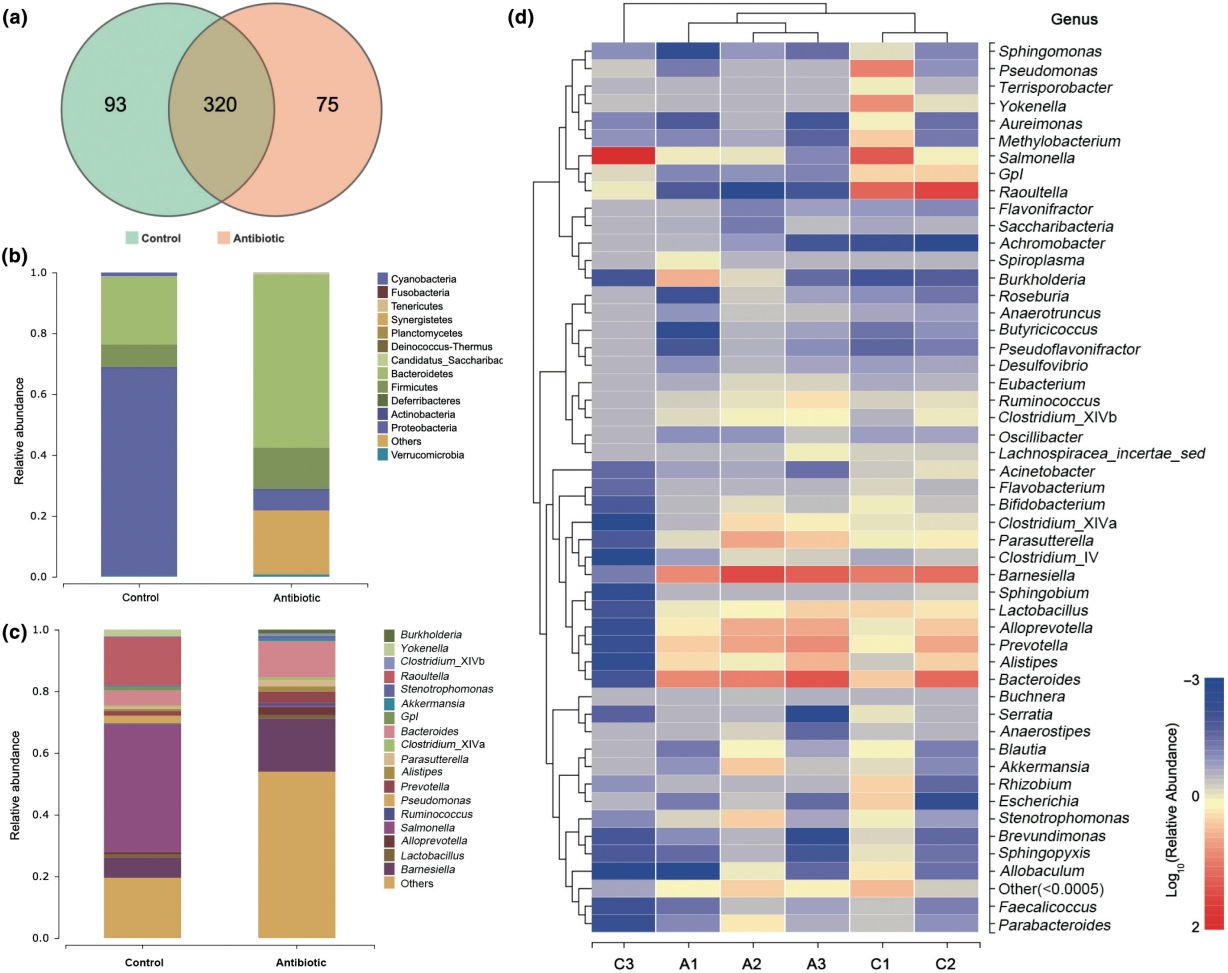
(a) The Venn diagram shows the number of shared and specific OTUs from the C and A groups. Average relative abundances of gut bacteria at the phylum (b) and genus (c) levels (Taxa with abundances <1% are included in “others”). (d) Heatmap clustering analysis of the gut bacteria at the genus level.

In the present study, we obtained a total of 386,148 clean reads with high quality from the normal‐ and antibiotic‐fed *A. viridicyanea* guts after quality control using 16S V3‐V4 rRNA amplicon sequencing. After alignment of these reads, a total of 488 OTUs were identified, and a Venn diagram of OTUs is shown in Figure 1a, suggesting that the two experimental groups shared 320 OTUs, and the C and A groups included 93 and 75 unique OTUs, respectively. Furthermore, the dominant taxa of the C group at the genus level was *Salmonella*, *Raoultella*, *Barnesiella*, and *Bacteroides*, while *Barnesiella* and *Bacteroides* were the dominant taxa in the A group, and the abundances of *Salmonella* and *Raoultella* were decreased (Figure 1c). Likewise, the gut bacteria of *A. viridicyanea* at the phylum level indicated that Proteobacteria and Bacteroidetes mainly existed in the C group, and the top 2 in the A group were Bacteroidetes and Firmicutes (Figure 1b). In addition, the most abundant genera and the hierarchical clusters of the gut bacteria are shown in the heatmap (Figure 1d).

**FIGURE 2:**
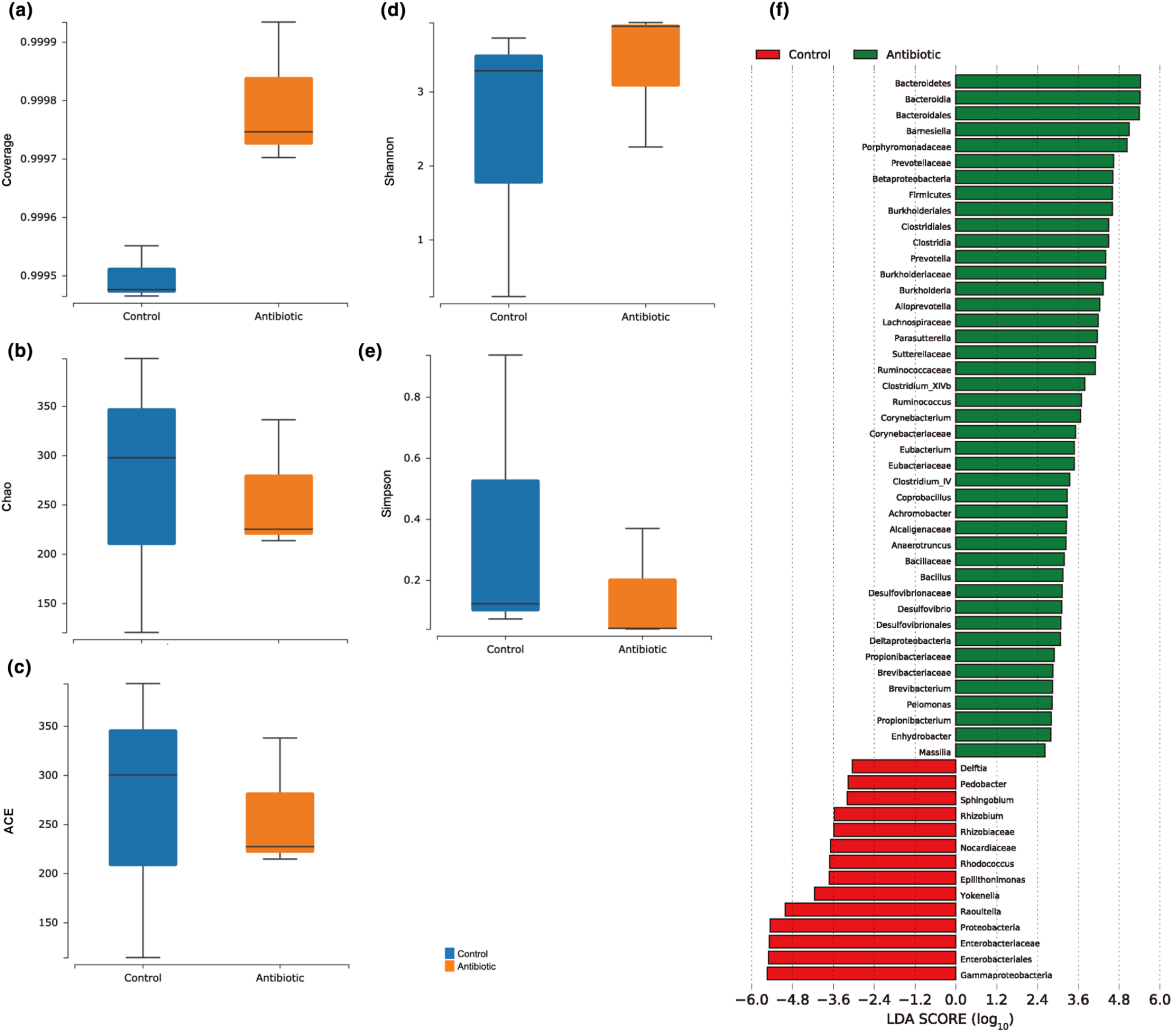
The alpha diversity of gut bacteria in *A. viridicyanea* from the C and A groups is estimated based on the number of OTUs, with coverage (a), Chao (b), ACE (c), Shannon (d), and Simpson (e) indices using Wilcox Test. (f) The taxa with significantly different abundances of gut bacteria of *A. viridicyanea* based on linear discriminate analysis (LDA) score from the C and A groups.

We further calculated the alpha diversity of the gut bacteria between the C and A groups in *A. viridicyanea*. As shown in Figure 2, the Goods coverage index was more than 0.99 in the C and A groups, and the Chao, ACE, and Simpson indices in the C group were higher than those in the A group. Overall, the abundance and alpha diversity of gut bacteria between these two samples were altered, but the difference was not significant (*p* > .05). Moreover, the gut bacterial communities obtained from the six sequencing groups were significantly clustered into two different groups in the PCoA plot (Figure S1). Next, linear discriminant analysis (LDA) effect size (LEfSe) analysis was used to detect discriminating taxa between the C and A groups (*p* < .05 and LDA > 2), indicating that the gut bacteria of *A. viridicyanea* in the antibiotic‐fed group was represented by some biomarkers. Specifically, the taxa *Bacteroideres*, *Bacteroidia*, and *Barnesiella* were significantly enriched in the A group, and *Delftia*, *Pedobacter*, and *Sphingobium* were significantly enriched in the C group (Figure 2f).

**FIGURE 3:**
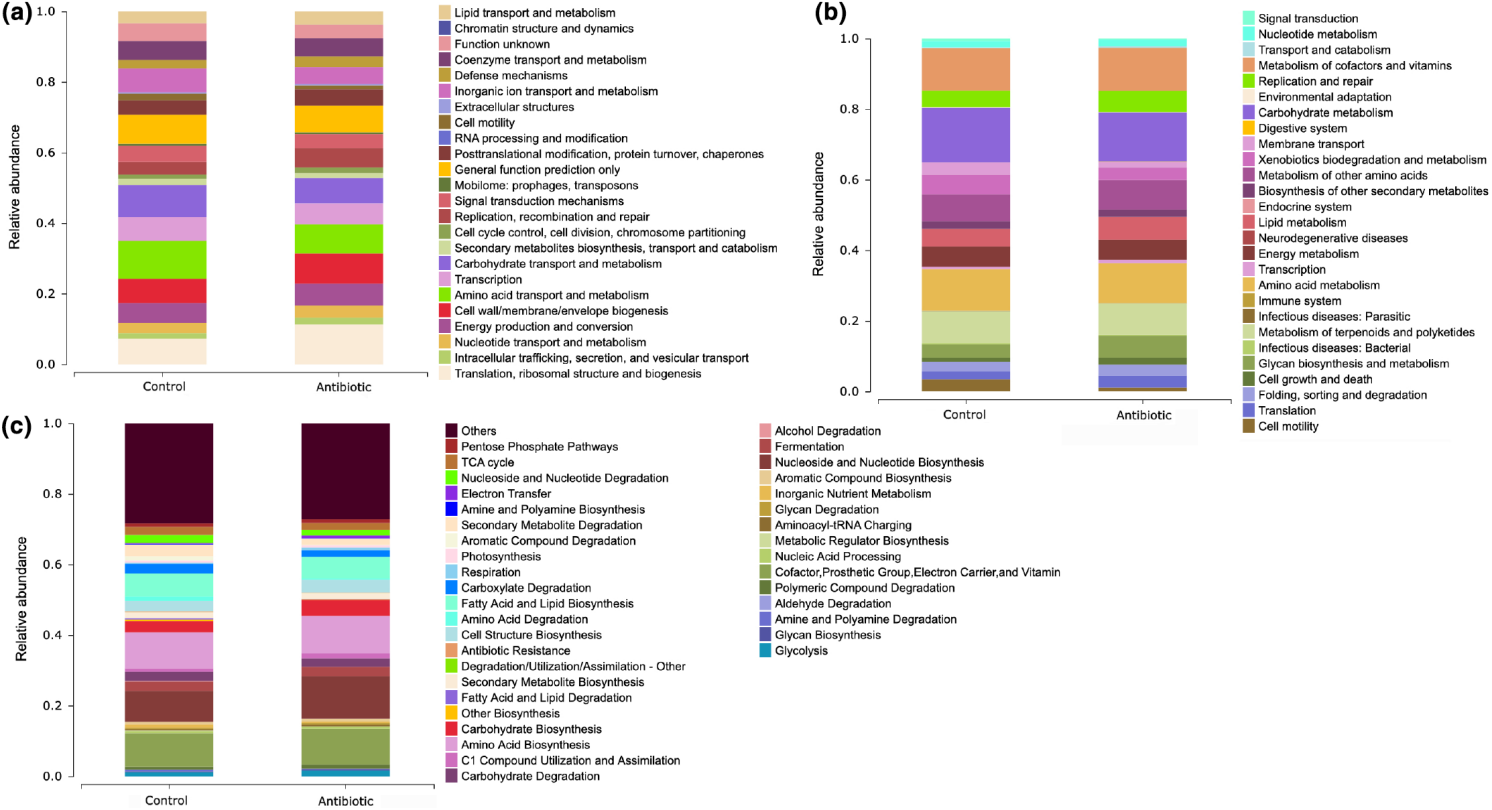
The functional classifications of gut bacteria in *A. viridicyanea* using COG (a), KEGG (b) and MetaCyc (c) databases. All the *x*‐axes represent the two experimental groups, and the *y*‐axes represent the relative abundance. Various colors indicate the term names in different databases.

Finally, to comprehensively illustrate the function of gut bacterial community in *A. viridicyanea* under normal and antibiotic treatments, we used the COG, KEGG, and Metacyc databases to classify and predict functions (Tables [Link], [Link]). The data implied that translation, ribosomal structure, and biogenesis was the maximum functional classifications after antibiotic‐fed treatment using COG enrichment analysis (Figure 3a). Carbohydrate metabolism, metabolism of cofactors and vitamins, and amino acid metabolism were the top 3 predicted pathways by KEGG annotation (Figure 3b), and nucleoside and nucleotide biosynthesis, amino acid biosynthesis, cofactor, prosthetic group, electron carrier, vitamin biosynthesis, and fatty acid and lipid biosynthesis were the main metabolic functions predicted by Metacyc annotation (Figure 3c). All these predicted functions may represent the most critical functions of the gut bacteria, and they played an essential role in host homeostasis.

### 3.2 Data analysis of small RNA sequencing

In this study, six sRNA‐seq libraries from the normal‐ and antibiotic‐fed *A. viridicyanea* guts generated a total of 102,216,241 (C group) and 94,961,924 (A group) raw reads, which were filtered to 98,807,924 and 92,356,655 clean reads, respectively, with average Q20 and Q30 scores of more than 98.97% and 96.87% (Table 1). The resulting lengths of clean reads are shown in Figure S2, ranging from 18 to 32 nt, suggesting that the miRNA length range is 18–24 nt, piRNA length range is 26–32 nt, and siRNA length range is 20–25 nt. In this research, we further focused on the identification, expression, and function of miRNAs.

**TABLE 1:** Quality assessment of sRNA‐seq data.

Sample ID	Raw reads	Total clean reads	Low quality reads rate (%)	Invalid adapter reads rate (%)	Read length too small rate (%)	Clean reads Q20 (%)	Clean reads Q30 (%)
A1	34,949,085	33,956,294	0.53	1.86	0.45	99	96.9
A2	35,174,514	34,197,516	0.53	1.9	0.34	99	97
A3	24,838,325	24,202,845	0.56	1.91	0.09	98.9	96.7
C1	35,006,838	34,070,692	0.54	1.92	0.21	99	97
C2	32,727,362	31,543,718	0.55	2.66	0.41	99	96.9
C3	34,482,041	33,193,514	0.55	2.5	0.68	98.9	96.7

### 3.3 Identification of known and novel miRNAs

The results revealed that the mapping ratio of clean reads exceeded 86.49% in each sRNA‐seq group by mapping onto the *A. viridicyanea* genome (Table S4). Moreover, all clean reads were compared to known sRNAs with various databases, indicating that a total of 122,766 and 168,803 reads were mapped to sRNAs in the A and C groups, respectively, showing that 39 sRNAs were regarded as rRNAs and tRNAs identified. Herein, we identified a total of 2220 miRNAs, including 44 known miRNAs and 2176 novel miRNAs, with U (uridine) bias at the first nucleotide position and G (guanine) at the least frequent nucleotide (data not shown).

### 3.4 Expression patterns of miRNAs

**FIGURE 4:**
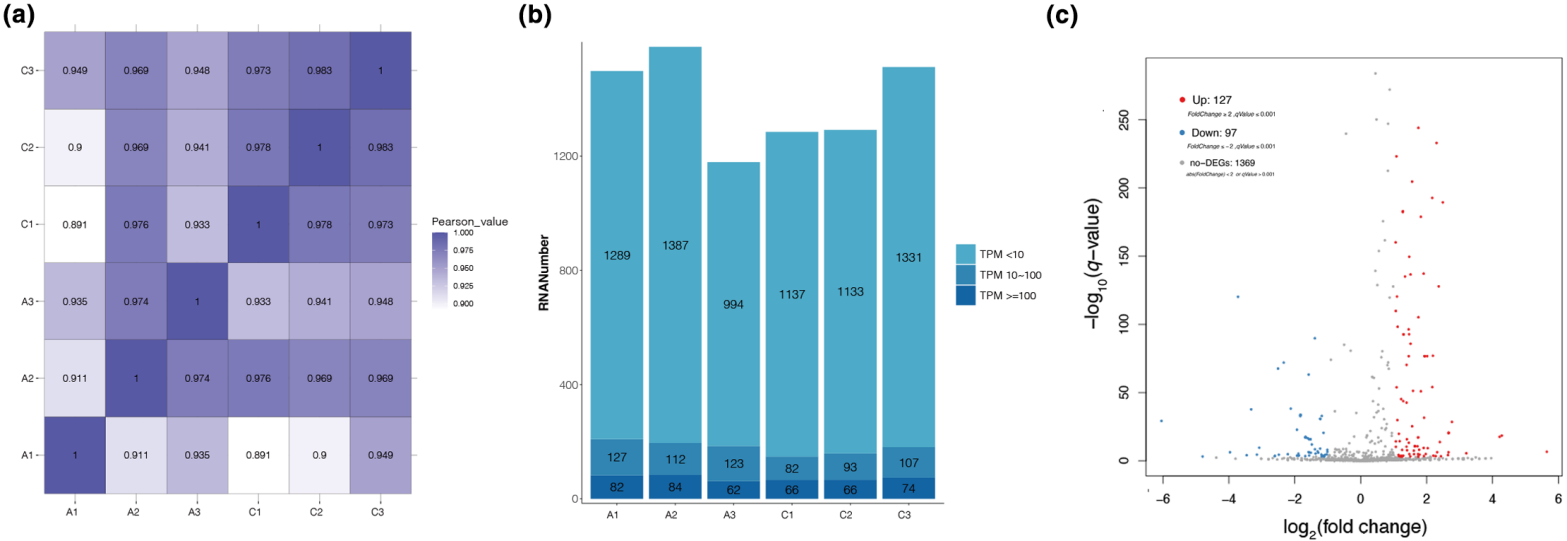
(a) Correlation heatmap of sRNA‐seq among the six sRNA‐seq groups. (b) The expression levels of all miRNAs among the six sRNA‐seq groups. (c) Volcano plot of DEMs between the C and A groups. Each point in this figure represents one miRNA. The *x*‐axis indicates the gene expression with fold change (antibiotics vs. control), and the *y*‐axis indicates the *p* values adjusted by the false discovery rate (FDR; *q* value) levels.

Principal component analysis (PCA) revealed strong aggregation of all sRNA‐seq samples (data not shown), and besides, the *R* ^2^ values (Pearson correlation coefficients) were 0.891–1.000 among the six sRNA‐seq groups based on FPKM values, representing the robustness of the biological replicates and reliability of the sRNA‐seq data (Figure 4a). Furthermore, as shown in Figure 4b, the TPM values of most miRNAs were less than 10 among the six sRNA‐seq groups. The gut tissues of antibiotic‐fed individuals were used as experimental groups, and normally reared samples were used as controls, suggesting that a total of 224 miRNAs were screened as significantly differentially expressed miRNAs under the condition of *p* value <.001 and log_2_ | fold change | >1, of which 127 miRNAs were upregulated and 97 miRNAs were downregulated (Figure 4c and Figure S3). Finally, seven DEMs were randomly selected to qRT‐PCR validation, revealing that the expression levels of DEMs by qRT‐PCR validation were consistent with the sRNA‐seq data (Figure S4).

### 3.5 Target prediction and functional annotation

**FIGURE 5:**
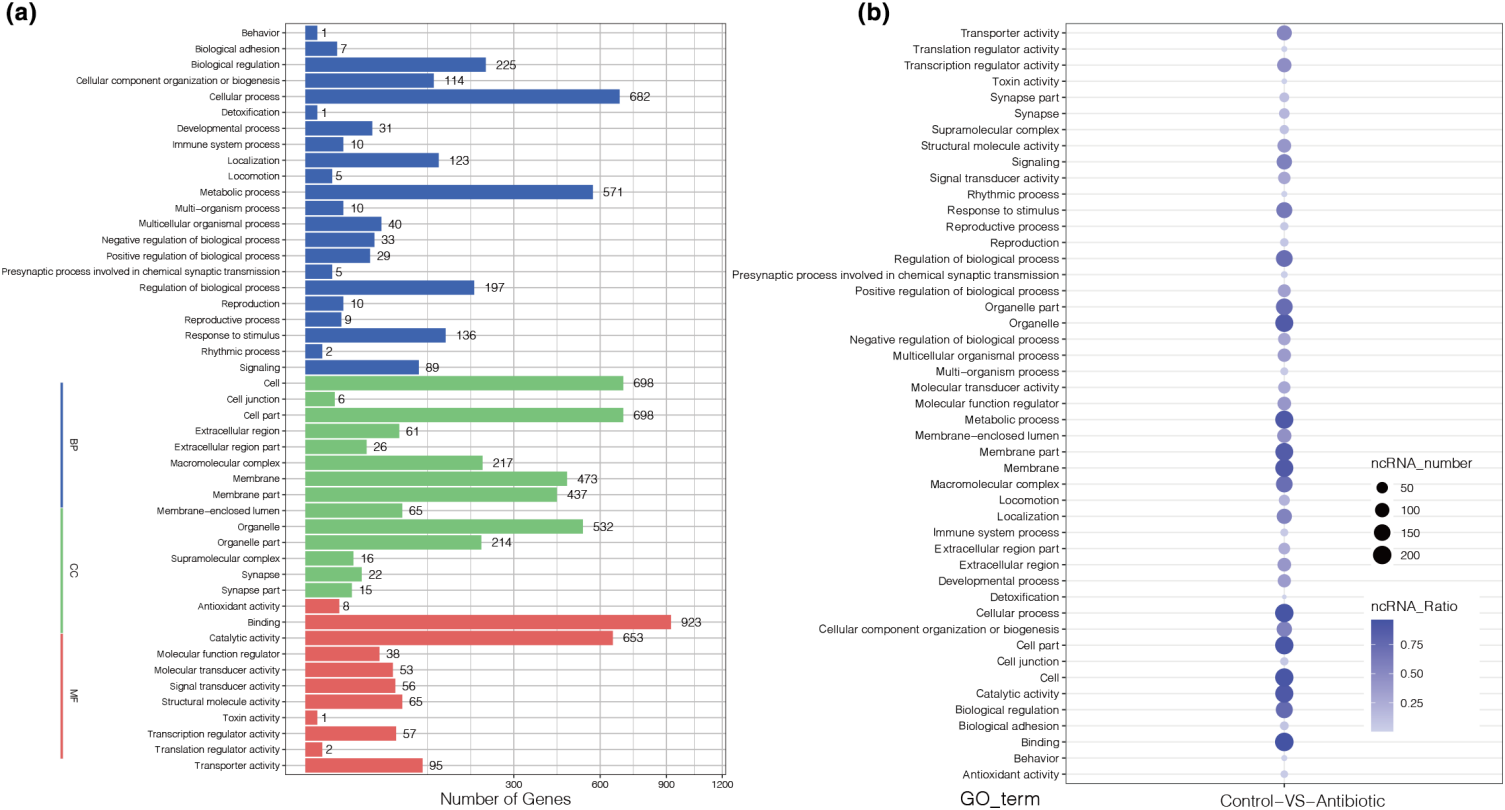
(a) GO enrichment of target genes. Each bar represents the gene number in each GO term in the category of BP (biological process), CC (cellular component) and MF (molecular function). The *x*‐axis indicates the number of genes. (b) GO enrichment of DEMs. A larger circle indicates a greater number of enriched DEMs. The *x*‐axis indicates the comparison group. All the *y*‐axes are the GO functional classifications.

To explore the functional roles of miRNAs and target genes, we identified 2014 miRNAs targeting 11,395 genes, including 224 DEMs targeting 10,594 genes. The results demonstrated that target genes were involved in a wide range of biological functions, including biological process (BP), cellular component (CC), and molecular function (MF) by GO annotation. In detail, 47 GO terms were assigned to target genes; of these terms, 22 terms belonged to biological process, 14 terms belonged to cellular component, and 11 terms belonged to molecular function, including binding, followed by cell, cell part, cellular process, catalytic activity, and so forth (Figure 5a and Table S5). As shown in Figure 5b, DEMs were mainly enriched in binding, cellular process, cell, cell part, and catalytic activity. Notably, 12 DEMs were enriched in immune system process, 136 DEMs were enriched in response to stimulus, and 81 DEMs were enriched in multicellular organismal process.

**FIGURE 6:**
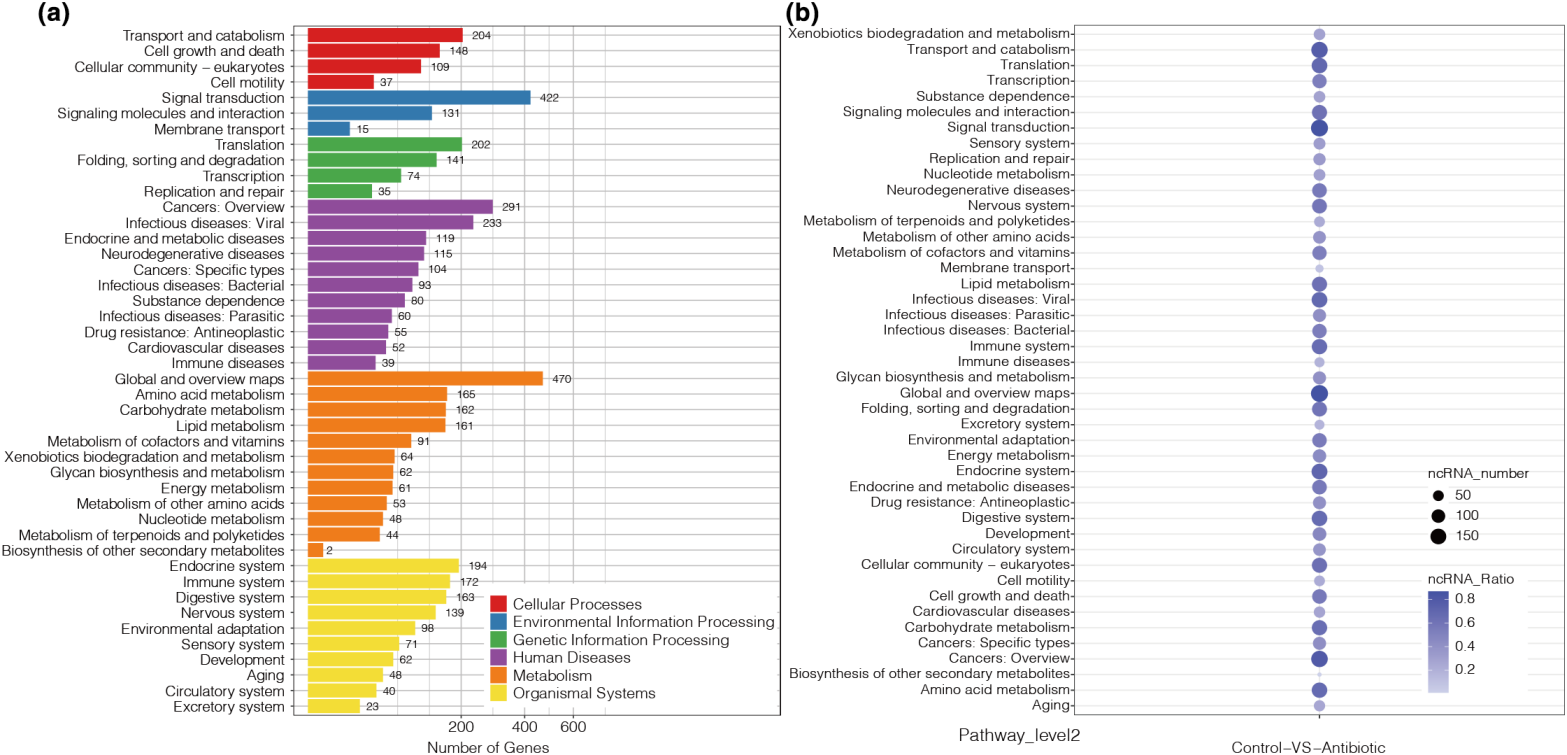
(a) KEGG enrichment of target genes. Plot diameter represents the numbers of target genes in a KEGG classification. The *x*‐axis indicates the number of genes. According to KEGG classification, the main pathways can be divided into five categories: Cellular processes (red), environmental information processing (blue), genetic information processing (green), human diseases (purple), metabolism (orange), and organismal systems (yellow). (b) KEGG enrichment of DEMs. A larger circle indicates a greater number of enriched DEMs. The *x*‐axis indicates the comparison group. All the *y*‐axes are the KEGG functional classifications.

To clarify the functional pathways of DEMs and target genes, the KEGG enrichment results suggested that a total of 5152 target genes were annotated to 44 KEGG pathway subcategories from six categories, including cellular processes, environmental information processing, genetic information processing, human diseases, metabolism, and organismal systems (Figure 6, Table S6). The dominant pathways of target genes were global and overview maps, signal transduction, cancers: overview, and infectious diseases: viral. Furthermore, we identified and summarized numerous DEMs from various immune and metabolism‐related pathways, such as the Toll/Imd signaling pathway, Toll‐like receptor signaling pathway, NOD‐like receptor signaling pathway, RIG‐I‐like receptor signaling pathway, NF‐κB signaling pathway, fatty acid metabolism, metabolism of xenobiotics by cytochrome P450, and so forth (Table 2). In summary, these results indicated that the annotation information of DEMs and target genes could lay the foundation for further in‐depth studies to characterize their roles in insect homeostasis.

**TABLE 2:** Summary of DEMs in immune and metabolism‐related pathways.

Items	Pathway ID	Pathway name	DEM numbers
Immune pathways	ko04624	Toll/Imd signaling pathway	33
	ko04620	Toll‐like receptor signaling pathway	20
	ko04621	NOD‐like receptor signaling pathway	14
	ko04622	RIG‐I‐like receptor signaling pathway	12
	ko04657	IL‐17 signaling pathway	31
	ko04630	JAK/STAT signaling pathway	14
	ko04064	NF‐κB signaling pathway	13
	ko04151	PI3K/Akt signaling pathway	75
	ko04013	MAPK signaling pathway—fly	92
	ko00524	Neomycin, kanamycin and gentamicin biosynthesis	1
	ko04623	cytosolic DNA‐sensing pathway	36
	ko04142	Lysosome	103
Metabolism‐related pathways	ko00030	Pentose phosphate pathway	30
	ko00980	Metabolism of xenobiotics by cytochrome P450	30
	ko00650	Butanoate metabolism	19
	ko01200	Carbon metabolism	99
	ko04979	Cholesterol metabolism	31
	ko00270	Cysteine and methionine metabolism	52
	ko00471	D‐Glutamine and D‐glutamate metabolism	1
	ko00565	Ether lipid metabolism	1
	ko00071	Fatty acid metabolism	31
	ko00051	Fructose and mannose metabolism	12
	ko00052	Galactose metabolism	28
	ko00480	Glutathione metabolism	60
	ko00564	Glycerophospholipid metabolism	30

### 3.6 Interaction network of selected DEM and their target genes

**FIGURE 7:**
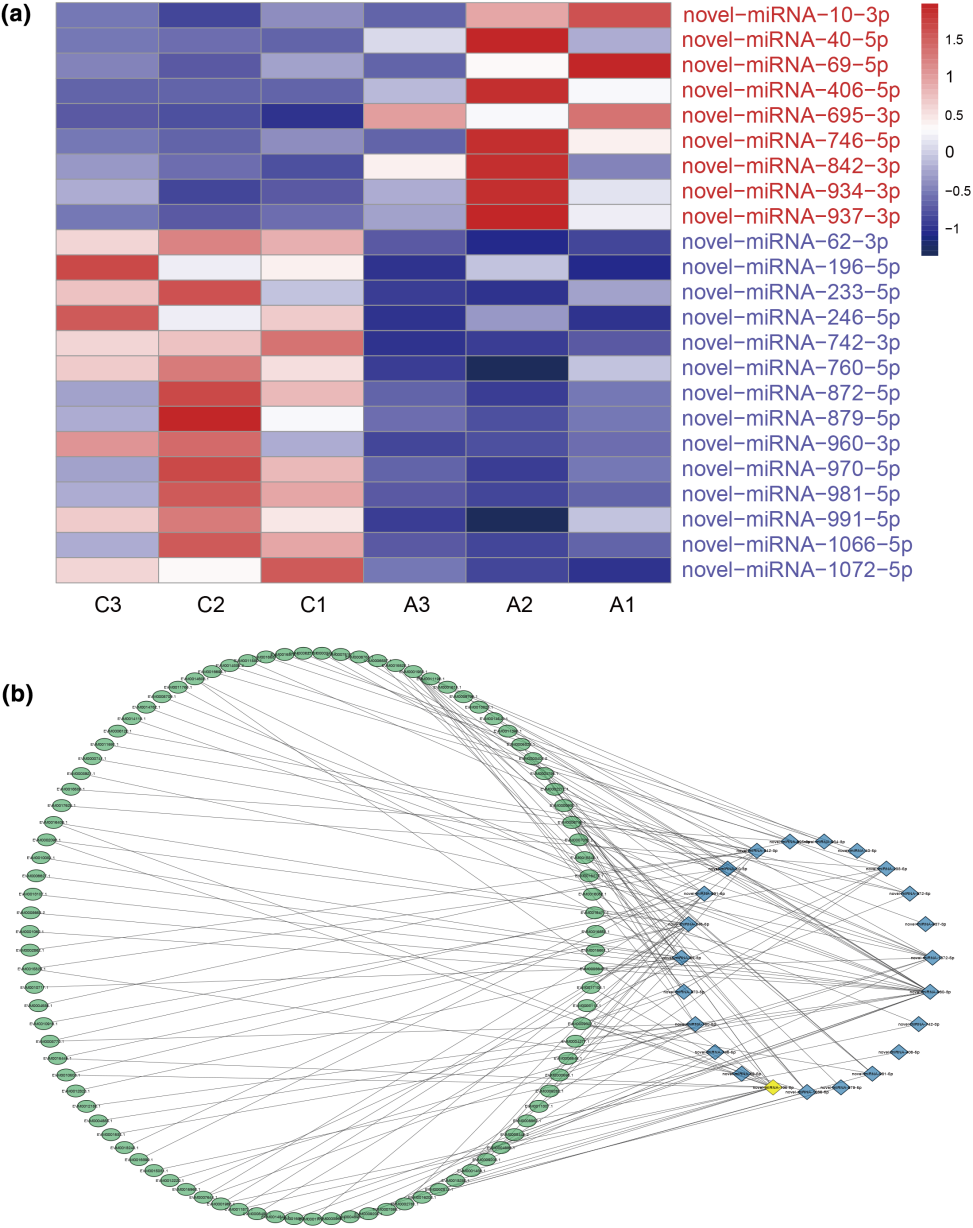
(a) Heatmap of 23 DEMs for constructing a regulatory network with FPKM values among the six sRNA‐seq groups. Red and blue colors represent upregulated and downregulated DEMs, respectively. (b) MiRNA‐mRNA regulatory network between selected DEMs and their target genes. Green ellipses indicate target genes, and blue and yellow diamonds indicate DEMs.

Based on the functional enrichments and target prediction, we randomly selected 23 DEMs and 133 target genes to construct a network map from four innate immune pathways, including Toll/Imd, JAK/STAT, MAPK‐fly, and NF‐κB signaling pathways, as well as four metabolism‐related pathways, including metabolism of xenobiotics by cytochrome P450, carbon metabolism, fatty acid metabolism, and glutathione metabolism. Concretely, nine upregulated DEMs and 14 downregulated DEMs were selected to build the interaction network (Figure 7a), and meanwhile, the regulatory information between selected DEMs and target genes revealed that novel‐miRNA‐960‐3p targeted 22 genes; novel‐miRNA‐246‐5p targeted 14 genes; novel‐miRNA‐1072‐5p targeted 10 genes; novel‐miRNA‐62‐3p and novel‐miRNA‐842‐3p targeted 9 genes; novel‐miRNA‐233‐5p targeted 7 genes; novel‐miRNA‐760‐5p and novel‐miRNA‐991‐5p targeted 6 genes; novel‐miRNA‐981‐5p and novel‐miRNA‐1066‐5p targeted 5 genes; novel‐miRNA‐872‐5p and novel‐miRNA‐970‐5p targeted 4 genes; novel‐miRNA‐695‐3p and novel‐miRNA‐10‐3p targeted 3 genes; novel‐miRNA‐742‐3p, novel‐miRNA‐879‐5p and novel‐miRNA‐746‐5p targeted 2 genes; and novel‐miRNA‐406‐5p, novel‐miRNA‐69‐5p, novel‐miRNA‐937‐3p and novel‐miRNA‐40‐5p targeted one gene. In addition, novel‐miRNA‐196‐5p (yellow diamond) was found to regulate only 15 target genes in the JAK/STAT signaling pathway but not those in metabolism‐related pathways (Figure 7b). This finding suggested that some DEMs could play multifunctional roles in the regulation of physical activities and provided new basis for further exploring the regulatory mechanisms based on the competing endogenous RNA (ceRNA) networks in *A. viridicyanea*.

### 3.7 Correlation analysis between selected DEMs and gut microbial changes

**FIGURE 8:**
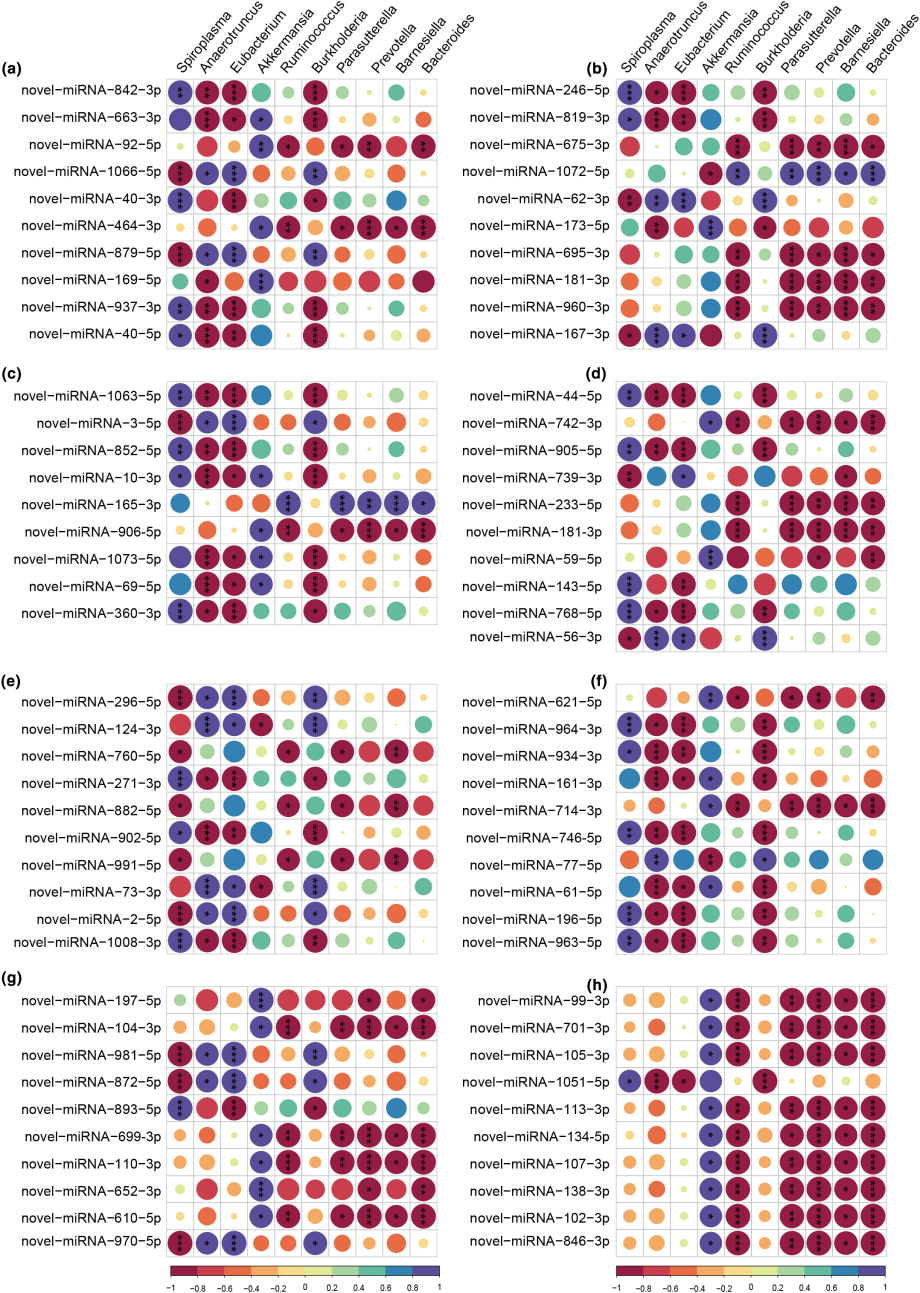
Correlation analyses between the gut bacteria and DEMs of immune or metabolism‐related pathways from the C and A groups. Each column represents different gut bacteria, and each row represents various DEMs. Red and blue colors represent negative or positive correlations, respectively. Ellipses sizes are proportional to correlation value, and correlation significance is shown by *** (*p* < .001), ** (*p* < .01) and * (*p* < .05). (a) Toll/Imd signaling pathway, (b) JAK/STAT signaling pathway, (c) MAPK‐fly signaling pathway, (d) NF‐κB signaling pathway, (e) metabolism of xenobiotics by cytochrome P450, (f) carbon metabolism, (g) fatty acid metabolism, and (h) glutathione metabolism.

To determine the correlation relationships between two sequencing data, we selected the top 10 abundant changes in gut bacteria and 10 DEMs from each signaling pathway (the selected pathways are similar to Section 3.6) using Pearson correlation analysis. At first, detailed information of randomly selected DEMs from eight pathways for correlation analysis is presented in Table S7, containing various upregulated or downregulated DEMs in each pathway. Moreover, the data is shown in Table S8 and Figure 8, demonstrating that *Bacteroides*, *Barnesiella*, *Prevotella*, *Parasutterella*, and *Ruminococcus* are not significantly positively correlated with DEMs in the Toll/Imd and NF‐κB signaling pathways, and they are less significantly associated with DEMs than those in the JAK/STAT and MAPK‐fly signaling pathways. Similarly, *Bacteroides*, *Barnesiella*, *Prevotella*, *Parasutterella*, and *Ruminococcus* were also not significantly positively associated with DEMs in four metabolism‐related pathways. Overall, the number of DEMs in eight pathways significantly negatively correlated with changes in the gut bacteria was larger than that with significantly positive correlation. Additionally, some DEMs, such as novel‐miRNA‐1066‐5p, novel‐miRNA‐879‐5p, novel‐miRNA‐981‐5p, novel‐miRNA‐872‐5p, and novel‐miRNA‐970‐5p, were conversely significantly association with different gut bacterial changes in same pathway.

## 4 DISCUSSION

There is accumulating evidence that the homeostasis between the insects and gut microbiota community was maintained within diverse biological processes (Kim & Lee, 2014). The gut microbiota contributes to effect on the insect's physiological traits at both the local (gut) and systemic (body) levels (Ma et al., 2021). Without this knowledge, the molecular mechanisms of host–microbiota interactions in non‐model insects could not be deeply elucidated. Here, we speculated that the dysbiosis of gut bacterial microbiota might affect miRNA expression to further regulate target genes using normal‐ and antibiotic‐fed *A. viridicyanea*, and it has been revealed that the gut bacterial alteration in beetles by antibiotic treatment resulted in a significant decrease in the body weight of newly emerged adults and prolonged development time (Wei et al., 2020). Collectively, our data mainly investigated the relationships between changes in the gut bacteria and DEMs and laid the foundation for the potential mechanisms for gut microbiota–insect interactions in Coleoptera.

Diverse gut bacteria in insects can significantly impact the host homeostasis (Douglas, 2015). For example, the larvae of *A. swainsoni* possess highly organized gut bacteria in four gut segments that benefit its host, including adapting to the relatively harsh niche conditions and playing a major role in host growth (Zhang, Zhuang, et al., 2022), so we aimed to get more detailed knowledge about the variation in diversity, composition and function of gut bacteria from normal‐ and antibiotic‐reared *A. viridicyanea*. Based on 16S rRNA amplicon sequencing, the number of sequences per sample was far greater than 60,000, and numerous OTUs were identified. The alpha diversity of gut bacteria from the normal samples was more abundant than that in the antibiotic‐fed groups, such as the Chao, ACE, and Simpson indices, but there were no significant differences among the two experimental groups, which is in agreement with our previous report (Wei et al., 2020). The composition of the gut bacterial community of *A. viridicyanea* was further investigated at the phylum level, with Firmicutes, Bacteroidetes, and Proteobacteria being the most dominant in the two experimental groups, which can affect a variety of aspects of host biology, such as nutritional absorption, insecticide resistance, and the degradation of defense chemicals (Ley et al., 2006; Salem et al., 2017; Zheng et al., 2023). Furthermore, the organization of gut bacterial community at the genus level was also observed, implying that *Salmonella* and *Raoultella*, as well as *Bacteroides* and *Barnesiella*, were most prevalent in the C and A samples, respectively. Mounting data indicated that they may be involved in inflammation, aging, and carbohydrate and short‐chain fatty acid metabolism (Borton et al., 2017; Fan et al., 2023; Li, Yang, et al., 2021; Zhao et al., 2023). Finally, we annotated the functional information of gut bacterial community from normal‐ and antibiotic‐fed *A. viridicyanea* when the results were compared with three databases, mainly including organic metabolism and biosynthesis. Likewise, it is well known that KEGG significantly enriched in the gut bacteria of *A. germari* and *A. swainsoni* contained biosynthesis and metabolism‐related pathways (Zhang, Wang, et al., 2022). All these results suggested that changes in the gut bacterial community by antibiotic treatment may affect the physiological biochemical action of host insects.

MiRNAs are one kind of sncRNAs that are approximately 22 nt in length, and they can influence immune responses, development, cell proliferation, and apoptosis in animals by base pairing with the target mRNA 3′‐untranslated regions (3′‐UTR) to trigger mRNA degradation or translational suppression (Gebert & MacRae, 2019; Li & Chen, 2021). In the current research, we constructed the six sRNA‐seq libraries from normal‐ and antibiotic‐fed *A. viridicyanea* guts to explore the miRNA profiles through next‐generation sequencing technology. SRNA‐seq data produced massive clean reads with high quality, and numerous sRNAs were screened, including miRNAs with approximately 18–24 nt and a dominant length of 22 nt, which was similar to previous reports (Chang et al., 2018; Wu, Jiang, et al., 2016; Wu, Qin, et al., 2016; Yang et al., 2022), piRNA with 26–32 nt in length, and siRNA with 20–25 nt in length. Later on, we identified a total of 2220 miRNAs, containing 44 known miRNAs and 2176 novel miRNAs, among which 224 DEMs were calculated between the A and C groups, consisting of 127 upregulated and 97 downregulated DEMs. Moreover, target gene prediction was performed with RNAhybrid and miRanda, illustrating that ten thousand target genes were obtained in this study.

The GO database provided useful information for understanding gene functions and specific processes, indicating 923 target genes in binding, followed by 698 target genes in cell and cell part, 682 target genes in cellular process, and 653 target genes in catalytic activity. Furthermore, total number of 203 DEMs were classified in binding, followed by 199 DEMs in cellular process, 196 DEMs in cell and cell part, and 191 DEMs in catalytic activity and metabolic process, which is consistent with the results in other insects (Wu et al., 2013; Wu, Jiang, et al., 2016; Wu, Qin, et al., 2016). Moreover, we also found that target genes and DEMs involved in various signaling pathways, including immune processes and metabolism, according to KEGG enrichment. As we known, insects can activate the humoral response, for example, the production of antimicrobial peptides by Toll/Imd (immune deficiency) and downstream signaling pathways, lysozymes, and rapidly activated phenoloxidase (PO) cascade‐mediated melanization constitute the humoral immune response of insects, and besides, they also initiate the cellular response to phagocytose bacteria and encapsulate parasites (Lemaitre & Hoffmann, 2007; Myllymaki et al., 2014; Sheehan et al., 2018). In *Drosophila*, the immune responses against gram‐positive bacteria and fungi mainly depend on the Toll pathway, whereas the Imd pathway has more function in defense against gram‐negative bacteria by the NF‐κB signaling pathway (Choe et al., 2005; Lemaitre et al., 1995; Leulier et al., 2002). In addition, there are two parallel pathways in the *Drosophila* gut, the DUOX pathway, and Imd pathway, which play indispensable roles in controlling the immune response and maintaining homeostasis in fruit flies (Bosco‐Drayon et al., 2012; Ha et al., 2005). So far, a growing number of reports have revealed miRNA roles in the modulation of the *Drosophila* Toll pathway. For example, miR‐8, miR‐958, miR‐964, and miR‐317 appear to downregulate the fly Toll pathway by combining with various genes (Choi & Hyun, 2012; Lee & Hyun, 2014; Li et al., 2019; Li, Li, et al., 2017; Li, Xu, et al., 2017). In previous data, in vivo experiments demonstrated a requirement for Ras/MAPK signaling in restricting innate immune responses in hemocytes, fat bodies, and adult gut stem cells in *Drosophila* (Ragab et al., 2011). The PI3K/Akt signaling pathway was significantly enriched with KEGG enrichment analysis, and upregulated genes from this pathway were identified in *Holotrichia parallela*, and moreover, silencing PI3K resulted in increasing susceptibility to entomopathogenic nematode and *Bacillus thuringiensis* stimulation and accelerating symbiotic bacterial multiplication (Li, Wu, et al., 2023). Here, our study provided more valuable data for the regulation between miRNA and immune pathways in non‐model insects. In addition to immune function, miRNAs have impact on the host metabolic systems, for instance, glutathione metabolism and metabolism of xenobiotics by cytochrome P450 contain cytochrome P450 family genes and glutamate and glutamine genes, which play important roles in the transcriptional regulation of hypoxia, maintaining normal immune system function and antioxidant effects in animals (Saetan et al., 2020). On the basis of the above observations, to better understand the regulatory relationships of DEMs and target genes, a co‐expression network was constructed according to enrichment data and target prediction, showing that numerous genes could be potentially regulated by different DEMs in eight signaling pathways, of note, the novel miRNA‐196‐5p might play a certain function in innate immune regulation in the *A. viridicyanea* gut, and further, their function should be deeply investigated in the future.

To our knowledge, the gut bacteria of insects can play important roles in homeostasis to improve host fitness by supplying essential amino acids and nutrients and affecting host immunity against invading pathogens, for example, some hemipteran insects have to abandon some innate immune systems, including the AMPs and Imd pathways, and thus they supply the essential amino acids and nutrients and protections by gut bacteria (Hill & Artis, 2010; Ma et al., 2022). Therefore, it is possible that the gut bacterial dynamic changes by antibiotic treatment could help us to interpret adaptive evolution in insects. Honey bees (*A. mellifera*), with high economic value and ecosystem importance, was observed that its gut bacteria might influence host health by modulating immune responses (Kwong et al., 2017). Wild populations of *Spodoptera frugiperda* are faced with various environmental stresses, such as pathogens or pesticides, and different diets, thereby they increase the abundance of the gut bacteria and partial immune system to improve environmental adaptation (Zheng et al., 2023). Similarly, Duplouy et al. (2020) altered the gut bacteria of the Glanville fritillary butterfly (*Melitaea cinxia*, L.) through antibiotic treatments, leading to upregulation of an antimicrobial peptide. Besides, many reports have validated that the Imd pathway mediates many aspects of the actions of gut bacteria such as *Acetobacter pomorum* and *Lactobacillus plantarum*, which play significant roles in promoting both immune and nonimmune functions through the Imd immune system in *Drosophila* (Zhai et al., 2018). In the present study, the association of gut bacterial changes from 16S rRNA amplicon sequencing and DEMs from sRNA‐seq indicated that different gut bacteria at the genus level significantly positively or negatively associated with miRNA profiles in immune‐ and metabolism‐related pathways. At first, combined with our previous study, we found a classical Toll/Imd‐MAPK/NF‐κB axis that contains upregulated and downregulated DEMs and target genes in response to changes in the gut bacteria of *A. viridicyanea* (Ren et al., 2023). We further observed that gut bacterial shifts also affected the expression changes of metabolism‐related miRNAs, implying that gut bacteria are most likely linked to miRNA profiles, which could play significant roles in adaptive evolution and maintaining homeostasis of *A. viridicyanea*.

In conclusion, this study provides the first data on genome‐wide identification and characterization of miRNAs and investigates changes in the gut bacteria of *A. viridicyanea* under normal‐ and antibiotic‐reared condition. The differentially expressed miRNAs between the two experimental samples are expected to offer new evidences to explore the biological functions between DEMs and target genes. Finally, high correlations between shifts in the gut bacteria and several DEMs were calculated and presented by Pearson correlation analysis, which can lay the foundation for uncovering the adaptive evolution by gut bacteria–host interactions in Coleoptera. Further works are necessary to validate the function of the related miRNAs and target genes based on the ceRNA networks in *A. viridicyanea*.

## AUTHOR CONTRIBUTIONS

**Yipeng Ren:** Conceptualization (equal); formal analysis (equal); investigation (equal); methodology (equal); supervision (equal); visualization (equal); writing – original draft (lead); writing – review and editing (equal). **Yuan Wang:** Investigation (equal); methodology (equal); resources (equal); writing – review and editing (equal). **Juhong Chen:** Data curation (equal); investigation (equal); methodology (equal); resources (equal); software (equal); writing – review and editing (equal). **Siying Fu:** Data curation (equal); formal analysis (equal); investigation (equal); writing – review and editing (equal). **Wenjun Bu:** Project administration (equal); visualization (equal); writing – review and editing (equal). **Huaijun Xue:** Funding acquisition (equal); investigation (equal); project administration (equal); supervision (equal); writing – review and editing (equal).

## FUNDING INFORMATION

This work was financially supported by the National Natural Science Foundation of China (Grant Nos. 31672334 and 32170456) and the Fundamental Research Funds for the Central Universities (Grant No. 63213120).

## CONFLICT OF INTEREST STATEMENT

The authors declare no conflict of interest.

## PERMISSION TO REPRODUCE MATERIALS FROM OTHER SOURCES

None.

## Supporting information


Figure S1.

Figure S2.

Figure S3.

Figure S4.

Table S1.

Table S2.

Table S3.

Table S4.

Table S5.

Table S6.

Table S7.

Table S8.

Table S9.

## Data Availability

The raw sequencing data in this study were deposited in the GenBank under accession number PRJNA946034 and PRJNA946198.

## References

[ece310660-bib-0001] Bai, J. Y. , Xu, Z. , Li, L. , Zhang, Y. , Diao, J. , Cao, J. , Xu, L. , & Ma, L. (2023). Gut bacterial microbiota of *Lymantria dispar asiatica* and its involvement in *Beauveria bassiana* infection. Journal of Invertebrate Pathology, 197, 107897. 36806463 10.1016/j.jip.2023.107897

[ece310660-bib-0002] Bartel, D. P. (2004). microRNAs: Genomics, biogenesis, mechanism, and function. Cell, 116(2), 281–297. 14744438 10.1016/s0092-8674(04)00045-5

[ece310660-bib-0003] Borton, M. A. , Sabag‐Daigle, A. , Wu, J. , Solden, L. M. , O'Banion, B. S. , Daly, R. A. , Wolfe, R. A. , Gonzalez, J. F. , Wysocki, V. H. , Ahmer, B. M. M. , & Wrighton, K. C. (2017). Chemical and pathogen‐induced inflammation disrupt the murine intestinal microbiome. Microbiome, 5(1), 47. 28449706 10.1186/s40168-017-0264-8 PMC5408407

[ece310660-bib-0004] Bosco‐Drayon, V. , Poidevin, M. , Boneca, I. G. , Narbonne‐Reveau, K. , Royet, J. , & Charroux, B. (2012). Peptidoglycan sensing by the receptor PGRP‐LE in the *Drosophila* gut induces immune responses to infectious bacteria and tolerance to microbiota. Cell Host & Microbe, 12, 153–165. 22901536 10.1016/j.chom.2012.06.002

[ece310660-bib-0005] Caporaso, J. G. , Kuczynski, J. , Stombaugh, J. , Bittinger, K. , Bushman, F. D. , Costello, E. K. , Fierer, N. , Peña, A. G. , Goodrich, J. K. , Gordon, J. I. , Huttley, G. A. , Kelley, S. T. , Knights, D. , Koenig, J. E. , Ley, R. E. , Lozupone, C. A. , McDonald, D. , Muegge, B. D. , Pirrung, M. , … Knight, R. (2010). QIIME allows analysis of highthroughput community sequencing data. Nature Methods, 7(5), 335–336. 20383131 10.1038/nmeth.f.303 PMC3156573

[ece310660-bib-0006] Chang, Z. X. , Akinyemi, I. A. , Guo, D. Y. , & Wu, Q. (2018). Characterization and comparative analysis of microRNAs in the rice pest *Sogatella furcifera* . PLoS ONE, 13(9), e0204517. 30248141 10.1371/journal.pone.0204517 PMC6152972

[ece310660-bib-0007] Chen, X. , & Rosbash, M. (2017). microRNA‐92a is a circadian modulator of neuronal excitability in *Drosophila* . Nature Communications, 8, 14707. 10.1038/ncomms14707 PMC5347142 28276426

[ece310660-bib-0008] Choe, K. M. , Lee, H. , & Anderson, K. V. (2005). *Drosophila* peptidoglycan recognition protein LC (PGRP‐LC) acts as a signal‐transducing innate immune receptor. Proceedings of the National Academy of Sciences of the United States of America, 102, 1122–1126. 15657141 10.1073/pnas.0404952102 PMC545828

[ece310660-bib-0009] Choi, I. K. , & Hyun, S. (2012). Conserved microRNA miR‐8 in fat body regulates innate immune homeostasis in *Drosophila* . Developmental and Comparative Immunology, 37, 50–54. 22210547 10.1016/j.dci.2011.12.008

[ece310660-bib-0010] Chu, C. C. , Spencer, J. L. , Curzi, M. J. , Zavala, J. A. , & Seufferheld, M. J. (2013). Gut bacteria facilitate adaptation to crop rotation in the western corn rootworm. Proceedings of the National Academy of Sciences of the United States of America, 110, 11917–11922. 23798396 10.1073/pnas.1301886110 PMC3718123

[ece310660-bib-0011] Douglas, A. E. (2015). Multiorganismal insects: Diversity and function of resident microorganisms. Annual Review of Entomology, 60, 17–34. 10.1146/annurev-ento-010814-020822 PMC4465791 25341109

[ece310660-bib-0012] Duplouy, A. , Minard, G. , & Saastamoinen, M. (2020). The gut bacterial community affects immunity but not metabolism in a specialist herbivorous butterfly. Ecology and Evolution, 10, 8755–8876. 32884655 10.1002/ece3.6573 PMC7452788

[ece310660-bib-0013] Edgar, R. C. (2013). UPARSE: Highly accurate OTU sequences from microbial amplicon reads. Nature Methods, 10(10), 996–998. 23955772 10.1038/nmeth.2604

[ece310660-bib-0014] Egan, S. , & Gardiner, M. (2016). Microbial dysbiosis: Rethinking disease in marine ecosystems. Frontiers in Microbiology, 7, 991. 27446031 10.3389/fmicb.2016.00991 PMC4914501

[ece310660-bib-0015] Enright, A. J. , John, B. , Gaul, U. , Tuschl, T. , Sander, C. , & Marks, D. S. (2003). microRNA targets in *Drosophila* . Genome Biology, 5, R1. 14709173 10.1186/gb-2003-5-1-r1 PMC395733

[ece310660-bib-0016] Fan, Y. , Ju, T. T. , Bhardwaj, T. , et al. (2023). Week‐old chicks with high *Bacteroides* abundance have increased short‐chain fatty acids and reduced markers of gut inflammation. Microbiology Spectrum, 11, e0361622. 10.1128/spectrum.03616-22 36719194 PMC10100795

[ece310660-bib-0018] Gebert, L. F. R. , & MacRae, I. J. (2019). Regulation of microRNA function in animals. Nature Reviews. Molecular Cell Biology, 20(1), 21–37. 30108335 10.1038/s41580-018-0045-7 PMC6546304

[ece310660-bib-0019] Ha, E. M. , Oh, C. T. , Bae, Y. S. , & Lee, W. J. (2005). A direct role for dual oxidase in *Drosophila* gut immunity. Science, 310, 847–850. 16272120 10.1126/science.1117311

[ece310660-bib-0020] Hammer, T. J. , & Bowers, M. D. (2015). Gut microbes may facilitate insect herbivory of chemically defended plants. Oecologia, 179, 1–14. 25936531 10.1007/s00442-015-3327-1

[ece310660-bib-0021] Hill, D. A. , & Artis, D. (2010). Intestinal bacteria and the regulation of immune cell homeostasis. Annual Review of Immunology, 28, 623–667. 10.1146/annurev-immunol-030409-101330 PMC5610356 20192812

[ece310660-bib-0023] Kim, S. H. , & Lee, W. J. (2014). Role of DUOX in gut inflammation: Lessons from *Drosophila* model of gut‐microbiota interactions. Frontiers in Cellular and Infection Microbiology, 3, 116. 24455491 10.3389/fcimb.2013.00116 PMC3887270

[ece310660-bib-0024] Kozomara, A. , Birgaoanu, M. , & Griffiths‐Jones, S. (2019). MiRBase: From microRNA sequences to function. Nucleic Acids Research, 47, D155–D162. 30423142 10.1093/nar/gky1141 PMC6323917

[ece310660-bib-0025] Kwong, W. K. , Mancenido, A. L. , & Moran, N. A. (2017). Immune system stimulation by the native gut microbiota of honey bees. Royal Society Open Science, 4, 170003. 28386455 10.1098/rsos.170003 PMC5367273

[ece310660-bib-0026] Langmead, B. , Trapnell, C. , Pop, M. , & Salzberg, S. L. (2009). Ultrafast and memory‐efficient alignment of short DNA sequences to the human genome. Genome Biology, 10(3), R25. 19261174 10.1186/gb-2009-10-3-r25 PMC2690996

[ece310660-bib-0027] Lee, G. J. , & Hyun, S. (2014). Multiple targets of the microRNA miR‐8 contribute to immune homeostasis in *Drosophila* . Developmental and Comparative Immunology, 45, 245–251. 24694685 10.1016/j.dci.2014.03.015

[ece310660-bib-0028] Lee, J. , Cha, W. H. , & Lee, D. W. (2022). Multiple precursor proteins of thanatin isoforms, an antimicrobial peptide associated with the gut symbiont of *Riptortus pedestris* . Frontiers in Microbiology, 12, 796548. 35069496 10.3389/fmicb.2021.796548 PMC8767025

[ece310660-bib-0029] Legrand, T. P. , Wynne, J. W. , Weyrich, L. S. , & Oxley, A. P. (2020). A microbial sea of possibilities: Current knowledge and prospects for an improved understanding of the fish microbiome. Reviews in Aquaculture, 12(2), 1101–1134.

[ece310660-bib-0030] Lemaitre, B. , & Hoffmann, J. (2007). The host defense of *Drosophila melanogaster* . Annual Review of Immunology, 25, 697–743. 10.1146/annurev.immunol.25.022106.141615 17201680

[ece310660-bib-0031] Lemaitre, B. , Kromer‐Metzger, E. , Michaut, L. , Nicolas, E. , Meister, M. , Georgel, P. , Reichhart, J. M. , & Hoffmann, J. A. (1995). A recessive mutation, immune deficiency (imd), defines two distinct control pathways in the *Drosophila* host defense. Proceedings of the National Academy of Sciences of the United States of America, 92, 9465–9469. 7568155 10.1073/pnas.92.21.9465 PMC40822

[ece310660-bib-0032] Leulier, F. , Vidal, S. , Saigo, K. , Ueda, R. , & Lemaitre, B. (2002). Inducible expression of double‐stranded RNA reveals a role for dFADD in the regulation of the antibacterial response in *Drosophila* adults. Current Biology, 12, 996–1000. 12123572 10.1016/s0960-9822(02)00873-4

[ece310660-bib-0033] Lewis, B. P. , Burge, C. B. , & Bartel, D. P. (2005). Conserved seed pairing, often flanked by adenosines, indicates that thousands of human genes are microRNA targets. Cell, 120, 15–20. 15652477 10.1016/j.cell.2004.12.035

[ece310660-bib-0034] Ley, R. E. , Turnbaugh, P. J. , Klein, S. , & Gordon, J. I. (2006). Microbial ecology – Human gut microbes associated with obesity. Nature, 444(7122), 1022–1023. 17183309 10.1038/4441022a

[ece310660-bib-0035] Li, A. , Yang, Y. , Qin, S. , Lv, S. , Jin, T. , Li, K. , Han, Z. , & Li, Y. (2021). Microbiome analysis reveals gut microbiota alteration of early‐weaned Yimeng black goats with the effect of milk replacer and age. Microbial Cell Factories, 20, 78. 33789672 10.1186/s12934-021-01568-5 PMC8010993

[ece310660-bib-0036] Li, E. T. , Wu, H. J. , Wang, Z. M. , Li, K. B. , Zhang, S. , Cao, Y. Z. , & Yin, J. (2023). PI3K/Akt/CncC signaling pathway mediates the response to EPN‐Bt infection in *Holotrichia parallela* larvae. Pest Management Science, 79, 1660–1673. 10.1002/ps.7337 36565065

[ece310660-bib-0037] Li, I. , & Chen, Y. G. (2021). Emerging roles of circular RNAs in innate immunity. Current Opinion in Immunology, 68, 107–115. 33176221 10.1016/j.coi.2020.10.010 PMC7925352

[ece310660-bib-0038] Li, R. , Huang, Y. , Zhang, Q. , Zhou, H. , Jin, P. , & Ma, F. (2019). The miR‐317 functions as a negative regulator of toll immune response and influences *Drosophila* survival. Developmental and Comparative Immunology, 95, 19–27. 30708026 10.1016/j.dci.2019.01.012

[ece310660-bib-0039] Li, S. , Li, Y. , Shen, L. , Jin, P. , Chen, L. , & Ma, F. (2017). miR‐958 inhibits toll signaling and drosomycin expression via direct targeting of toll and Dif in *Drosophila melanogaster* . American Journal of Physiology. Cell Physiology, 312, C103–C110. 27974298 10.1152/ajpcell.00251.2016 PMC5336595

[ece310660-bib-0040] Li, S. , Wu, J. L. , Huo, Y. L. , Zhao, X. , & Xue, L. (2021). Profiling multiple heavy metal contamination and bacterial communities surrounding an iron tailing pond in Northwest China. Science of the Total Environment, 752, 141827. 32889271 10.1016/j.scitotenv.2020.141827

[ece310660-bib-0041] Li, S. , Xu, J. , Sun, L. , Li, R. , Jin, P. , & Ma, F. (2017). *Drosophila* miR‐964 modulates toll signaling pathway in response to bacterial infection. Developmental and Comparative Immunology, 77, 252–258. 28823799 10.1016/j.dci.2017.08.008

[ece310660-bib-0043] Livak, K. J. , & Schmittgen, T. D. (2001). Analysis of relative gene expression data using realtime quantitative PCR and the 2(T)(‐Delta Delta C) method. Methods, 25, 402–408. 11846609 10.1006/meth.2001.1262

[ece310660-bib-0044] Ma, L. , Liu, L. , Zhao, Y. , Yang, L. , Chen, C. , Li, Z. , & Lu, Z. (2020). JNK pathway plays a key role in the immune system of the pea aphid and is regulated by microRNA‐184. PLoS Pathogens, 16(6), e1008627. 32584915 10.1371/journal.ppat.1008627 PMC7343183

[ece310660-bib-0045] Ma, L. , Liu, S. L. , Lu, P. , Yan, X. , Hao, C. , Wang, H. , Wei, J. , Qie, X. , & Lu, Z. (2022). The IMD pathway in hemipteran: A comparative analysis and discussion. Developmental and Comparative Immunology, 136, 104513. 35977558 10.1016/j.dci.2022.104513

[ece310660-bib-0046] Ma, M. , Tu, C. , Luo, J. , Lu, M. , Zhang, S. , & Xu, L. (2021). Metabolic and immunological effects of gut microbiota in leaf beetles at the local and systemic levels. Integrative Zoology, 16(3), 313–323. 33704889 10.1111/1749-4877.12528

[ece310660-bib-0047] Myllymaki, H. , Valanne, S. , & Ramet, M. (2014). The *Drosophila* imd signaling pathway. Journal of Immunology, 192, 3455–3462. 10.4049/jimmunol.1303309 24706930

[ece310660-bib-0048] Otasek, D. , Morris, J. H. , Bouças, J. , Pico, A. R. , & Demchak, B. (2019). Cytoscape automation: Empowering workflow‐based network analysis. Genome Biology, 20, 185. 31477170 10.1186/s13059-019-1758-4 PMC6717989

[ece310660-bib-0049] Pertea, M. , Pertea, G. M. , Antonescu, C. M. , Chang, T. C. , Mendell, J. T. , & Salzberg, S. L. (2015). StringTie enables improved reconstruction of a transcriptome from RNA‐seq reads. Nature Biotechnology, 33, 290–295. 10.1038/nbt.3122 PMC4643835 25690850

[ece310660-bib-0050] Qu, A. , Bai, Y. L. , Wang, J. Y. , Bai, Y. , Wang, J. , Zhao, J. , Zeng, J. , Liu, Y. , Chen, X. , Ke, Q. , Jiang, P. , Zhang, X. , Li, X. , Xu, P. , & Zhou, T. (2023). Integrated mRNA and miRNA expression analyses for *Cryptocaryon irritans* resistance in large yellow croaker (*Larimichthys crocea*). Fish & Shellfish Immunology, 135, 108650. 36858330 10.1016/j.fsi.2023.108650

[ece310660-bib-0051] Ragab, A. , Buechling, T. , Gesellchen, V. , Spirohn, K. , Boettcher, A. L. , & Boutros, M. (2011). *Drosophila* Ras/MAPK signalling regulates innate immune responses in immune and intestinal stem cells. The EMBO Journal, 30, 1123–1136. 21297578 10.1038/emboj.2011.4 PMC3061042

[ece310660-bib-0052] Reid, C. A. , & Beatson, M. (2015). Disentangling a taxonomic nightmare: A revision of the Australian, Indomalayan and Pacific species of *Altica* Geoffroy, 1762 (Coleoptera: Chrysomelidae: Galerucinae). Zootaxa, 3918, 503–551. 25781108 10.11646/zootaxa.3918.4.3

[ece310660-bib-0053] Ren, Y. P. , Chen, J. H. , Wang, Y. , Fu, S. Y. , Bu, W. J. , & Xue, H. J. (2023). The lncRNA‐mediated ceRNA network of *Altica viridicyanea* is involved in the regulation of the toll/Imd signaling pathway under antibiotic treatment. Frontiers in Physiology, 14, 1244190. 37664435 10.3389/fphys.2023.1244190 PMC10470016

[ece310660-bib-0054] Robinson, M. D. , McCarthy, D. J. , & Smyth, G. K. (2010). edgeR: A Bioconductor package for differential expression analysis of digital gene expression data. Bioinformatics, 26, 139–140. 19910308 10.1093/bioinformatics/btp616 PMC2796818

[ece310660-bib-0055] Saetan, W. , Tian, C. , Yu, J. , Lin, X. , He, F. , Huang, Y. , Shi, H. , Zhang, Y. , & Li, G. (2020). Comparative transcriptome analysis of gill tissue in response to hypoxia in silver Sillago (*Sillago sihama*). Animals, 10(4), E628–E958. 10.3390/ani10040628 PMC7222756 32268576

[ece310660-bib-0056] Salem, H. , Bauer, E. , Kirsch, R. , Berasategui, A. , Cripps, M. , Weiss, B. , Koga, R. , Fukumori, K. , Vogel, H. , Fukatsu, T. , & Kaltenpoth, M. (2017). Drastic genome reduction in an herbivore's pectinolytic symbiont. Cell, 171(7), 1520–1531.e13. 29153832 10.1016/j.cell.2017.10.029

[ece310660-bib-0057] Segata, N. , Izard, J. , Waldron, L. , Gevers, D. , Miropolsky, L. , Garrett, W. S. , & Huttenhower, C. (2011). Metagenomic biomarker discovery and explanation. Genome Biology, 12, R60. 21702898 10.1186/gb-2011-12-6-r60 PMC3218848

[ece310660-bib-0058] Sekirov, I. , Russell, S. L. , Antunes, L. C. M. , & Finlay, B. B. (2010). Gut microbiota in health and disease. Physiological Reviews, 90, 859–904. 20664075 10.1152/physrev.00045.2009

[ece310660-bib-0059] Sheehan, G. , Garvey, A. , Croke, M. , & Kavanagh, K. (2018). Innate humoral immune defences in mammals and insects: The same, with differences ? Virulence, 9, 1625–1639. 30257608 10.1080/21505594.2018.1526531 PMC7000196

[ece310660-bib-0060] Thursby, E. , & Juge, N. (2017). Introduction to the human gut microbiota. The Biochemical Journal, 474(11), 1823–1836. 28512250 10.1042/BCJ20160510 PMC5433529

[ece310660-bib-0061] Wei, J. , Segraves, K. A. , Li, W. Z. , Yang, X. K. , & Xue, H. J. (2020). Gut bacterial communities and their contribution to performance of specialist Altica flea beetles. Microbial Ecology, 80, 946–959. 32880699 10.1007/s00248-020-01590-x

[ece310660-bib-0062] Wolf, J. B. (2013). Principles of transcriptome analysis and gene expression quantification: An RNA‐seq tutorial. Molecular Ecology Resources, 13(4), 559–572. 23621713 10.1111/1755-0998.12109

[ece310660-bib-0063] Wu, P. , Han, S. , Chen, T. , Qin, G. , Li, L. , & Guo, X. (2013). Involvement of microRNAs in infection of silkworm with *Bombyx mori* cytoplasmic polyhedrosis virus (BmCPV). PLoS ONE, 8, e68209. 23844171 10.1371/journal.pone.0068209 PMC3699532

[ece310660-bib-0064] Wu, P. , Jiang, X. , Guo, X. , Li, L. , & Chen, T. (2016). Genome‐wide analysis of differentially expressed microRNA in *Bombyx mori* infected with nucleopolyhedrosis virus. PLoS ONE, 11(11), e0165865. 27806111 10.1371/journal.pone.0165865 PMC5091789

[ece310660-bib-0065] Wu, P. , Qin, G. , Qian, H. , Chen, T. , & Guo, X. (2016). Roles of miR‐278‐3p in IBP2 regulation and *Bombyx mori* cytoplasmic polyhedrosis virus replication. Gene, 575, 264–269. 26348138 10.1016/j.gene.2015.09.009

[ece310660-bib-0066] Xie, J. F. , Cai, Z. H. , Zheng, W. P. , & Zhang, H. (2023). Integrated analysis of miRNA and mRNA expression profiles in response to gut microbiota depletion in the abdomens of female *Bactrocera dorsalis* . Insect Science, 30, 443–458. 35751912 10.1111/1744-7917.13091

[ece310660-bib-0067] Xue, H. J. , Li, W. Z. , Nie, R. E. , & Yang, X. K. (2011). Recent speciation in three closely related sympatric specialists: Inferences using multilocus sequence, post‐mating isolation and endosymbiont data. PLoS ONE, 6, e27834. 22110767 10.1371/journal.pone.0027834 PMC3217007

[ece310660-bib-0068] Xue, H. J. , Li, W. Z. , & Yang, X. K. (2014). Assortative mating between two sympatric closely‐related specialists: Inferred from molecular phylogenetic analysis and behavioral data. Scientific Reports, 4, 5436. 24961567 10.1038/srep05436 PMC4069675

[ece310660-bib-0069] Xue, H. J. , Magalhães, S. , Li, W. Z. , & Yang, X. K. (2009). Reproductive barriers between two sympatric beetle species specialized on different host plants. Journal of Evolutionary Biology, 22, 2258–2266. 19807830 10.1111/j.1420-9101.2009.01841.x

[ece310660-bib-0070] Xue, H. J. , Wei, J. N. , Magalhães, S. , Zhang, B. , Song, K. Q. , Liu, J. , Li, W. Z. , & Yang, X. K. (2016). Contact pheromones of 2 sympatric beetles are modified by the host plant and affect mate choice. Behavioral Ecology, 27, 895–902.

[ece310660-bib-0071] Yang, X. B. , Zhou, C. , Yang, J. P. , Gong, M. F. , Yang, H. , Long, G. Y. , & Jin, D. C. (2022). Identification and profiling of *Sogatella furcifera* microRNAs and their potential roles in regulating the developmental transitions of nymph–adult. Insect Molecular Biology, 31(6), 798–809. 35899838 10.1111/imb.12805

[ece310660-bib-0072] Yao, X. L. , Ni, J. J. , Lin, L. , Jin, P. , & Ma, F. (2023). The NF‐kB/relish activates miR‐308 to negatively regulate Imd pathway immune signaling in *Drosophila* . Journal of Immunology, 211, 591–600. 10.4049/jimmunol.2200680 37358278

[ece310660-bib-0073] Yuva‐Aydemir, Y. , Xu, X. L. , Aydemir, O. , Gascon, E. , Sayin, S. , Zhou, W. , Hong, Y. , & Gao, F. B. (2015). Downregulation of the host gene jigr1 by miR‐92 is essential for neuroblast self‐renewal in *Drosophila* . PLoS Genetics, 11(5), e1005264. 26000445 10.1371/journal.pgen.1005264 PMC4441384

[ece310660-bib-0074] Zhai, Z. Z. , Huang, X. S. , & Yin, Y. L. (2018). Beyond immunity: The Imd pathway as a coordinator of host defense, organismal physiology and behavior. Developmental and Comparative Immunology, 83, 51–59. 29146454 10.1016/j.dci.2017.11.008

[ece310660-bib-0075] Zhang, L. , Zhuang, T. , Hu, M. , Liu, S. , Wu, D. , & Ji, B. (2022). Gut microbiota contributes to lignocellulose deconstruction and nitrogen fixation of the larva of *Apriona swainsoni* . Frontiers in Physiology, 13, 1072893. 36620205 10.3389/fphys.2022.1072893 PMC9816477

[ece310660-bib-0076] Zhang, S. K. , Wang, Y. , Li, Z. K. , Xue, H. J. , Zhou, X. D. , & Huang, J. H. (2022). Two *Apriona* species sharing a host niche have different gut microbiome diversity. Molecular Ecology, 83, 1059–1072. 10.1007/s00248-021-01799-4 34302194

[ece310660-bib-0077] Zhang, Z. , Mu, X. , Cao, Q. , Shi, Y. , Hu, X. , & Zheng, H. (2022). Honeybee gut lactobacillus modulates host learning and memory behaviors via regulating tryptophan metabolism. Nature Communications, 13, 2037. 10.1038/s41467-022-29760-0 PMC9018956 35440638

[ece310660-bib-0078] Zhao, R. , Symonds, J. E. , Walker, S. P. , Steiner, K. , Carter, C. G. , Bowman, J. P. , & Nowak, B. F. (2023). Relationship between gut microbiota and Chinook salmon (*Oncorhynchus tshawytscha*) health and growth performance in freshwater recirculating aquaculture systems. Frontiers in Microbiology, 14, 1065823. 36825086 10.3389/fmicb.2023.1065823 PMC9941681

[ece310660-bib-0079] Zhao, Z. M. , Yin, H. T. , Shen, M. M. , Zhang, S. L. , Chen, Z. K. , Li, T. , Zhang, Z. D. , Zhao, W. G. , Guo, X. J. , & Wu, P. (2022). Transcriptome of miRNA during inhibition of *Bombyx mori* nuclear polyhedrosis virus by geldanamycin in BmN cells. Archives of Insect Biochemistry and Physiology, 110, e21880. 35191078 10.1002/arch.21880

[ece310660-bib-0080] Zheng, R. W. , Cheng, L. L. , Peng, J. , Li, Q. , Yang, F. , Yang, D. , Xia, Y. , & Tang, Q. (2023). Comparative analysis of gut microbiota and immune genes linked with the immune system of wild and captive *Spodoptera frugiperda* (Lepidoptera: Noctuidae). Developmental and Comparative Immunology, 138, 104530. 36084754 10.1016/j.dci.2022.104530

