# Supplementary figures and images for: Integrated analysis of miRNA profiles and gut bacterial changes in Altica viridicyanea following antibiotic treatment
Source: Ecol Evol. 2023 Oct 31;13(11):e10660. doi: 10.1002/ece3.10660 (PMC10616750; doi:10.1002/ece3.10660)

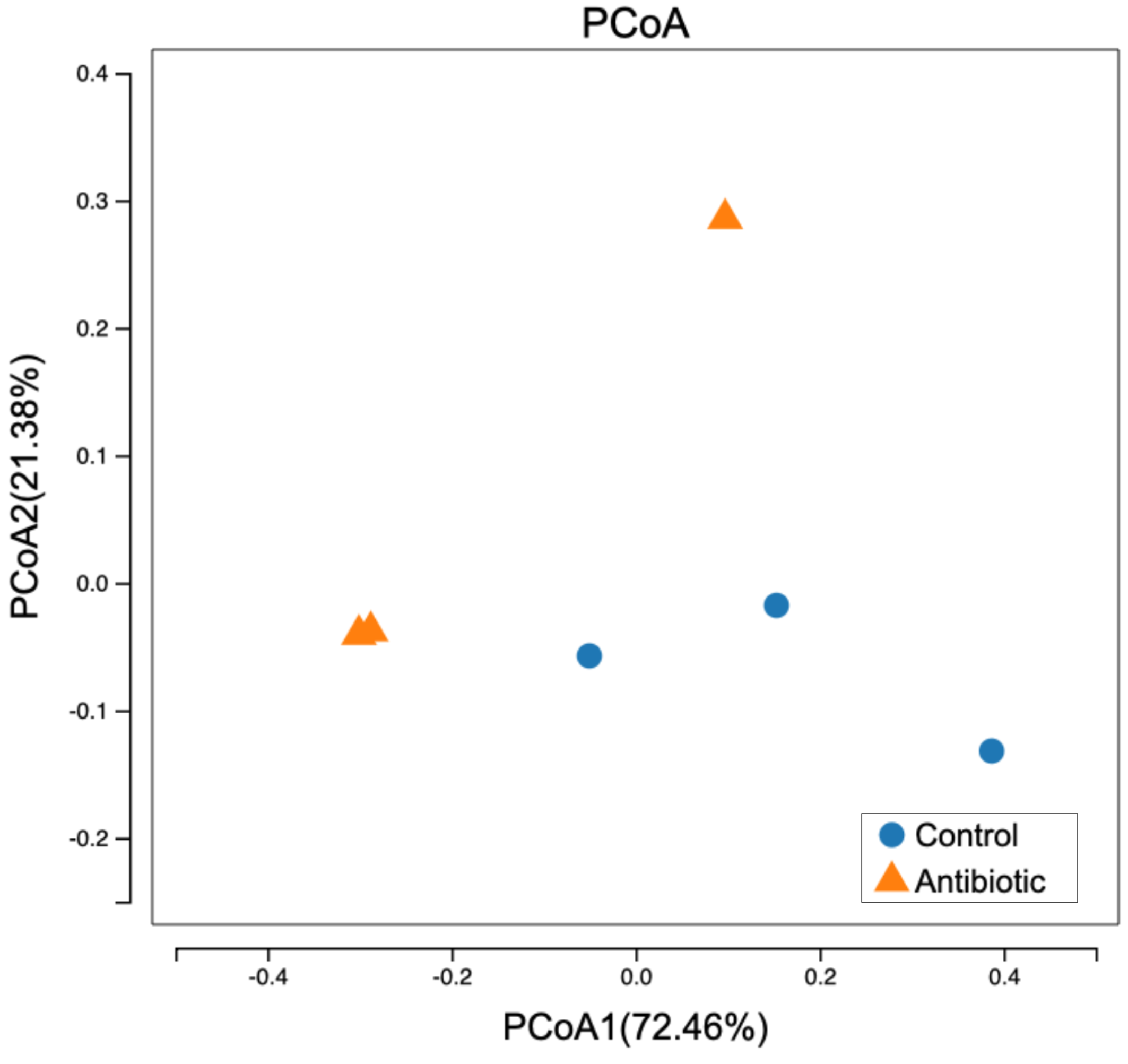

Supplement: Supplementary file 1 — Figure S1. [file ECE3-13-e10660-s002.tif]

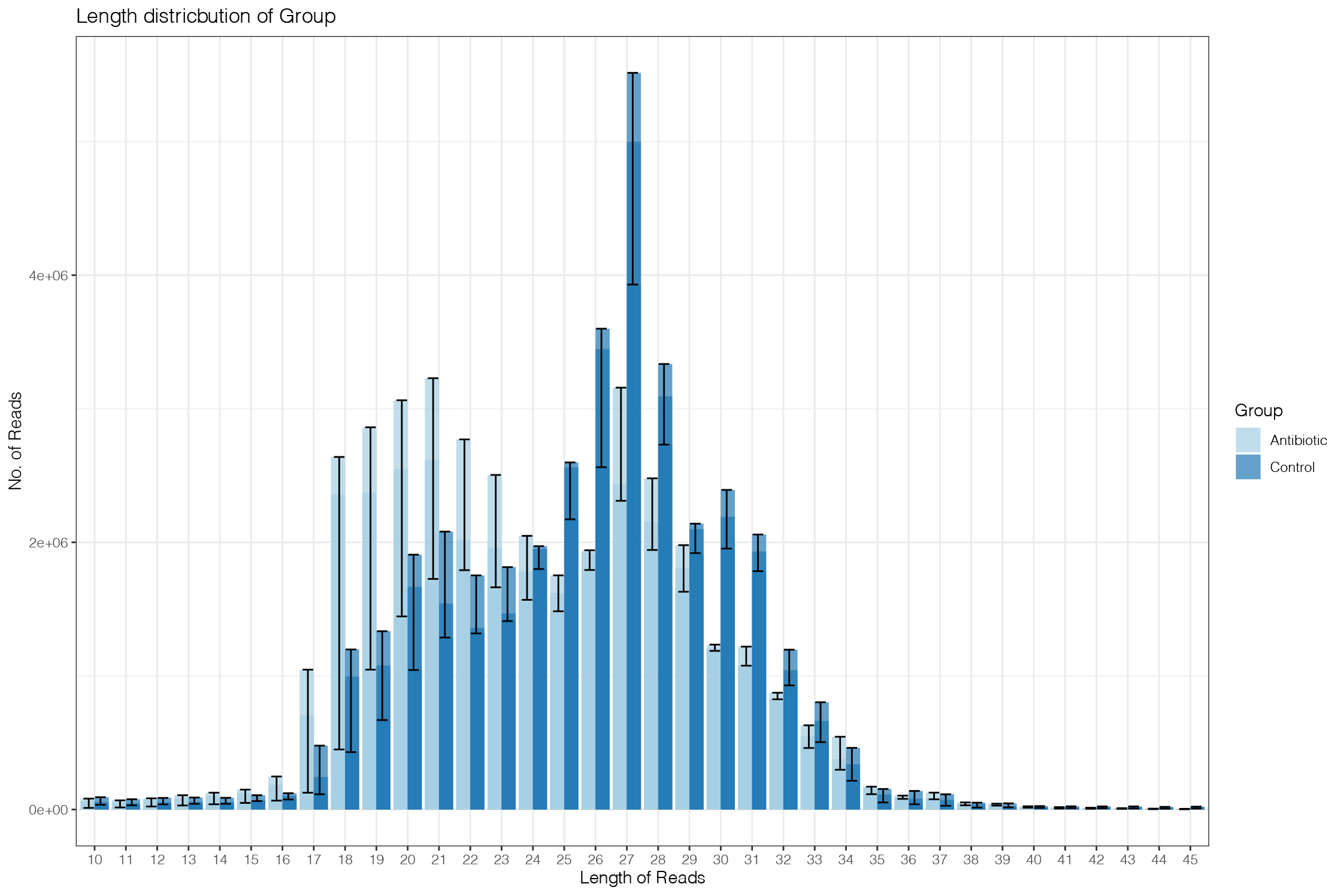

Supplement: Supplementary file 2 — Figure S2. [file ECE3-13-e10660-s005.tif]

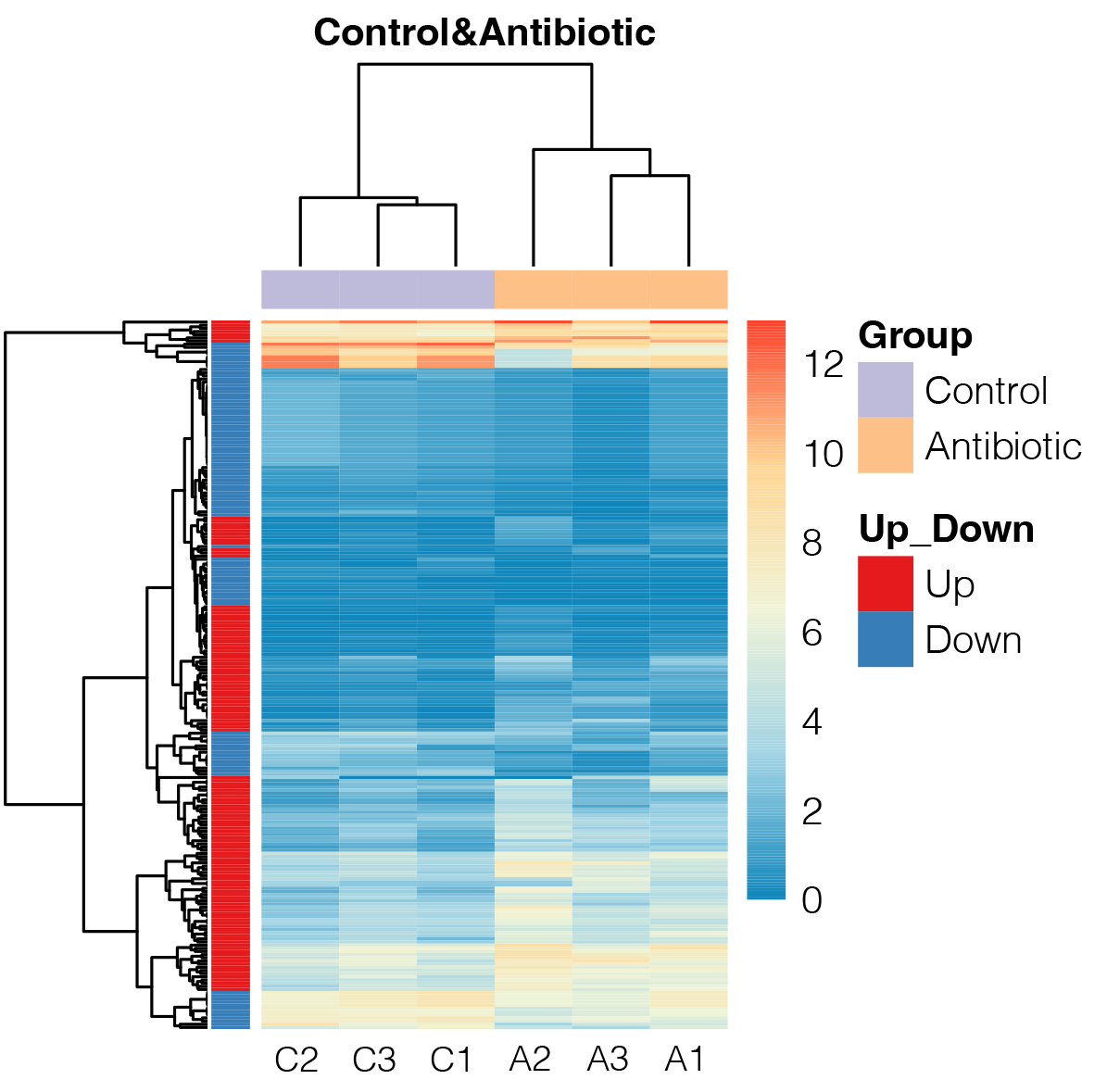

Supplement: Supplementary file 3 — Figure S3. [file ECE3-13-e10660-s009.tif]

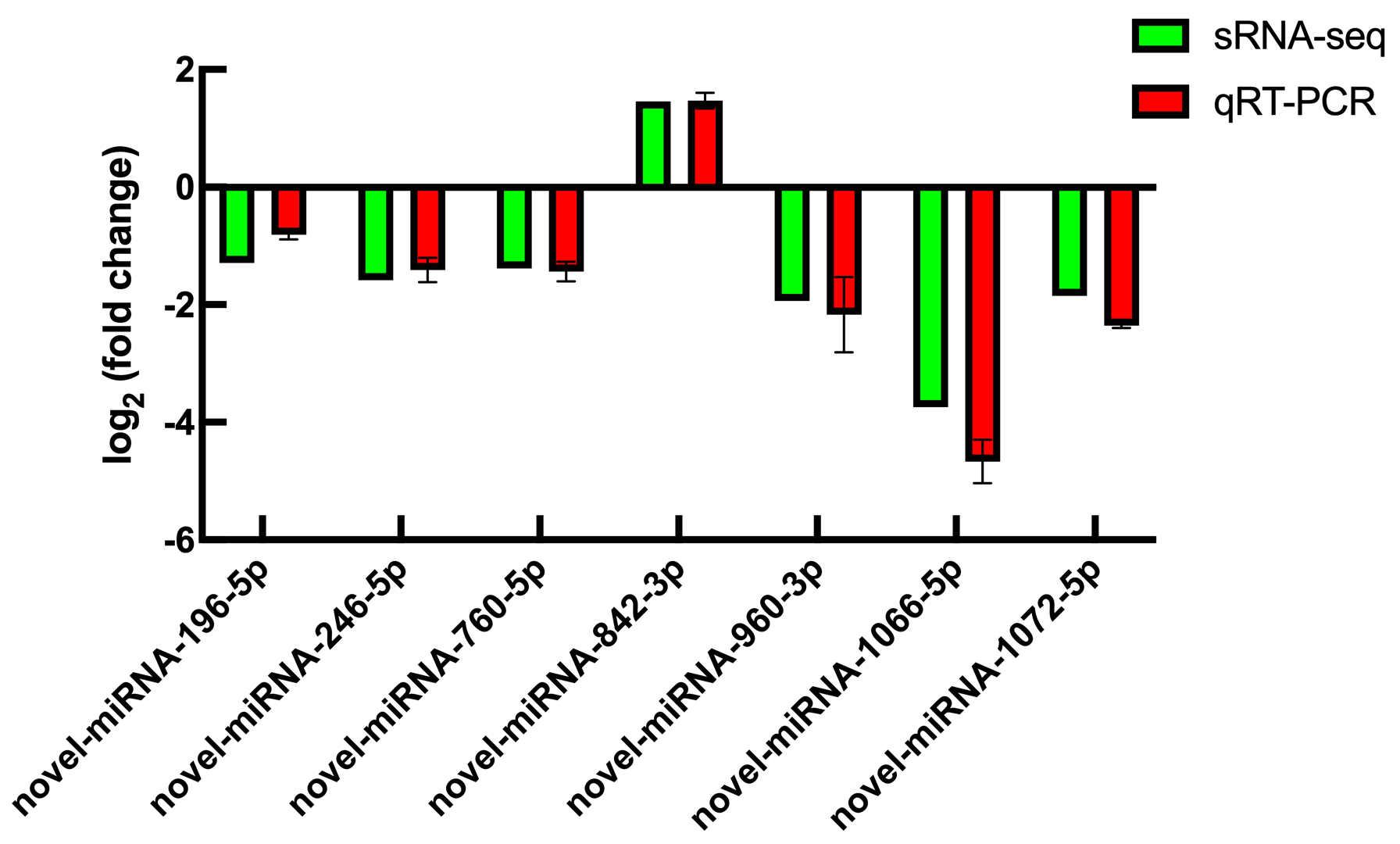

Supplement: Supplementary file 4 — Figure S4. [file ECE3-13-e10660-s011.tiff]
